# Immune microenvironment dynamics of HER2 overexpressing breast cancer under dual anti-HER2 blockade

**DOI:** 10.3389/fimmu.2023.1267621

**Published:** 2023-10-31

**Authors:** Sofia Batalha, Catarina Monteiro Gomes, Catarina Brito

**Affiliations:** ^1^ iBET, Instituto de Biologia Experimental e Tecnológica, Oeiras, Portugal; ^2^ Instituto de Tecnologia Química e Biológica António Xavier, Universidade Nova de Lisboa, Oeiras, Portugal

**Keywords:** HER2^+^ breast cancer, 3D cell models, trastuzumab, pertuzumab, tumor microenvironment, immunotherapies, immune response, NK cells

## Abstract

**Introduction:**

The clinical prognosis of the HER2-overexpressing (HER2-OE) subtype of breast cancer (BC) is influenced by the immune infiltrate of the tumor. Specifically, monocytic cells, which are promoters of pro-tumoral immunosuppression, and NK cells, whose basal cytotoxic function may be enhanced with therapeutic antibodies. One of the standards of care for HER2^+^ BC patients includes the combination of the anti-HER2 antibodies trastuzumab and pertuzumab. This dual combination was a breakthrough against trastuzumab resistance; however, this regimen does not yield complete clinical benefit for a large fraction of patients. Further therapy refinement is still hampered by the lack of knowledge on the immune mechanism of action of this antibody-based dual HER2 blockade.

**Methods:**

To explore how the dual antibody challenge influences the phenotype and function of immune cells infiltrating the HER2-OE BC microenvironment, we developed *in vitro* 3D heterotypic cell models of this subtype. The models comprised aggregates of HER2^+^ BC cell lines and human peripheral blood mononuclear cells. Cells were co-encapsulated in a chemically inert alginate hydrogel and maintained in agitation-based culture system for up to 7 days.

**Results:**

The 3D models of the HER2-OE immune microenvironment retained original BC molecular features; the preservation of the NK cell compartment was achieved upon optimization of culture time and cytokine supplementation. Challenging the models with the standard-of-care combination of trastuzumab and pertuzumab resulted in enhanced immune cytotoxicity compared with trastuzumab alone. Features of the response to therapy within the immune tumor microenvironment were recapitulated, including induction of an immune effector state with NK cell activation, enhanced cell apoptosis and decline of immunosuppressive PD-L1^+^ immune cells.

**Conclusions:**

This work presents a unique human 3D model for the study of immune effects of anti-HER2 biologicals, which can be used to test novel therapy regimens and improve anti-tumor immune function.

## Introduction

Breast cancer (BC) remains the deadliest female malignancy and is now the most frequently diagnosed form of cancer worldwide (1). About 20% of all BCs overexpress the human epidermal growth factor receptor 2 (HER2), a member of the epidermal growth factor receptor (EGFR) family (2). HER2^+^ BCs present poor outcomes (3), especially for the HER2-overexpressed (HER2-OE) BC surrogate intrinsic subtype. HER2-OE is characterized by the absence of estrogen and progesterone receptors and displays worse prognosis and survival rates than the Luminal B-like HER2^+^ subtype (4). In the last 20 years, anti-HER2 targeted therapies, namely the monoclonal antibody trastuzumab, led to expressive clinical improvement both for early-stage and metastatic patients (2, 5). Combination with further anti-HER2 blockade (the antibody pertuzumab, antibody-drug conjugates (ADCs), or tyrosine kinase inhibitors (TKis)) allowed bypassing the widespread acquired or inherent resistance to trastuzumab (2).

Trastuzumab and pertuzumab share the ability to engage the NK cell activating FcγRIIIA (CD16) receptor and induce specific killing of the opsonized HER2^+^ tumor cells via antibody-dependent cell-mediated cytotoxicity (ADCC), as demonstrated in several *in vitro* and *in vivo* studies (6–9). The HER2-OE subtype shows a high immune infiltration (10, 11), mostly composed by tumor infiltrating lymphocytes (TILs) and tumor-associated macrophages (TAMs) (12–18), which have been clinically associated with better (19, 20) and worse (21–23) prognosis, respectively. Specifically, the detection and function of the immune effectors CD8^+^ T cells and natural killer (NK) cells have been positively correlated not only with improved patient survival (18, 24–27) but also with response to therapeutic regimens, including trastuzumab (6, 7, 28, 29). On the other hand, patient data implicates breast cancer TAMs as the immune population with the highest expression of checkpoint ligand Programmed Death-Ligand 1 (PD-L1) (30), being responsible for direct suppression of immune effectors (23) and for the recruitment of peripheral immunosuppressive myeloid cells and T regulatory cells (T_Regs_) (22, 31–33). In fact, increased TAM infiltration (32, 34) and upregulation of PD-L1 in this cell population (32) were recently correlated with worse clinical response to trastuzumab-based therapy, supporting the link between patient outcome and immune effector status of the TME.

Remarkably, to date little is known about the dynamics of NK cell function in the tumor microenvironment (TME) of patients undergoing anti-HER2 blockade treatment. Despite the clinical success of the dual anti-HER2 blockade therapeutic strategies (2) and the known correlation between the TME and the response to therapy, the dynamics of the immune compartment during dual anti-HER2 treatment remain largely understudied. Increased infiltration of immune cells with trastuzumab treatment has been reported in patients (24, 26, 32, 35). There was an increase in the anti-tumoral NK and CD8^+^ T cell populations (24, 32, 35, 36), while tumor-promoting TAMs were reduced in patients who responded to neoadjuvant trastuzumab (32). On the dual blockade landscape, Griguolo et al. recently revisited data from the PAMELA trial to investigate changes in the immune compartment of HER2^+^ BC of patients undergoing treatment with trastuzumab and the TKi lapatinib, concluding that immune infiltration (including cytotoxic lymphocytes) is increased after two weeks of anti-HER2 treatment, in HER2-enriched subtypes that are hormone receptor negative (37).

Given the specific features of HER2-OE BC, namely the phenotype and behavior of the immune infiltrate and the patients’ response to anti-HER2 therapy, it is fundamental to develop reliable tools to study the immune modulation promoted by anti-HER2 targeting agents in an all-human setting. Three-dimensional (3D) culture systems can generate cancer models that resemble specific features of the TME architecture and composition and can be easily manipulated, interrogated, and generated in high throughput (38, 39). Probably due to the traditional view of BC as a poorly infiltrated tumor, 3D models for BC immune microenvironment are still limited, representing mostly the Luminal A (40–50) or the Triple Negative (40–42, 45–47, 51–57) clinicopathological surrogate subtypes. For HER2^+^ disease, the majority of the proposed 3D cell models with an immune component have been generated with BT474 cells (47, 49). This BC cell line retains expression of hormone receptors and therefore more closely represents the surrogate Luminal B subtype than the HER2-OE (58). As such, there is a need to develop 3D models of the tumor microenvironment including the immune compartment, which will more reliably represent the HER2-OE subtype.

The establishment of tumor-immune crosstalk in 3D models may be improved by increasing culture longevity and maintaining the spatial distribution of the different cell types (59), being essential to study the medium-to-long-term immune effects of drug challenge. Our team has pioneered culture modes coupling cell encapsulation in hydrogels and dynamic culture systems. These were shown to recreate native-like tumor-stromal interactions (60) and to allow long-term monitoring of the immune phenotype upon immunotherapy challenge (61). These strategies have not yet been applied to the study of HER2-OE BC crosstalk with the immune compartment (36).

This work presents a novel *in vitro* model of HER2-OE BC immune microenvironment, based on 3D heterotypic cultures composed of human HER2^+^ BC cell lines and human immune cells derived from peripheral blood mononuclear cells (PBMCs). Co-encapsulation of cells in alginate droplets promoted close contact between cell populations while avoiding the use of undefined animal-based matrices. This strategy allowed the preservation of tumor cell viability for at least one week via agitation-based culture, sustaining an efficient diffusion of soluble factors and oxygen. Refinement of co-culture time and cytokine supplementation were critical to preserve the NK cells, which are fundamental for the innate immune cytotoxicity mediated by anti-HER2 antibodies (62). The significance of the proposed models was highlighted in proof-of-concept challenges with the clinical standard-of-care combination of trastuzumab and pertuzumab. A comprehensive characterization of the immune microenvironment revealed the recapitulation of known features of TME response to trastuzumab-based therapy. The addition of pertuzumab could enhance NK cell activation, downregulate PD-L1 in immune cells and sharply decrease the monocytic population of the TME of HER2-OE BC.

## Materials and methods

### Cell culture

The HER2^+^ BC cell lines SKBR3 (ATCC, #HTB-30) and HCC1954 (ATCC, #CRL-2338) were cultured, respectively, in McCoy’s 5A Modified culture medium (Sigma-Aldrich, #M9309) supplemented with 10% fetal bovine serum (FBS, Gibco, #10270106), and in RPMI 1640 culture medium, no phenol red (Gibco, #11835105) supplemented with 10% FBS, 50µM β-Mercaptoetanol (Gibco, #31350010) and 6mM HEPES (Gibco, #15630056). The 2D cultures were kept at 37° C, in 5% CO_2_, and passaged at sub-confluency until passage number 29. Cell banks were routinely tested for mycoplasma contamination.

### Cell aggregation

SKBR3 cells were plated as a single cell suspension at a concentration of 2.1x10^6^ cell/well in Aggrewell™ 400 6-well plates (Stemcell Technologies), previously treated with anti-adherence rinsing solution (Stemcell Technologies), as per manufacturer’s instructions. Plates were centrifuged at 100 x *g* and cultured at 37° C for 4 days. HCC1954 cells were aggregated as previously described (63, 64). Briefly, cells were inoculated as a single cell suspension into 125 mL spinner vessels with flat centered cap and angled side arms (Corning), at a concentration of 2x10^5^ cell/mL, and cultured at an agitation rate of 80 rpm, for 4 days, to induce cell aggregation.

### Immune cell isolation

Peripheral blood mononuclear cells (PBMCs) were isolated from buffy coats of healthy donors obtained from the Portuguese Blood and Transplantation Institute (IPST) according to the Portuguese and European legislation and the commonly internationally accepted ethics directives and guidelines, including signed consent from the donors upon blood donation. Buffy coats were diluted in Isolation Buffer (phosphate buffer saline (PBS) containing 2% (v/v) FBS and 2 mM EDTA), layered on top of Lymphoprep™ (Stemcell Technologies), and centrifuged at 950 x *g* for 25 min. The white PBMC-containing interface layer was collected, washed with Isolation Buffer, and incubated with ACK Lysis Buffer (Gibco, #A1049201) to remove residual erythrocytes. PBMCs were cryopreserved in 10% DMSO (Sigma-Aldrich)/90% FBS, in the vapor phase of liquid nitrogen storage, until further use.

NK cells were isolated from cryopreserved PBMCs by negative selection through magnetic separation using EasySep™ Human NK cell Isolation Kit (Stemcell Technologies, #17955), according to manufacturer’s protocol, and used immediately after isolation.

### Microencapsulated 3D cultures

SKBR3 or HCC1954 aggregates were encapsulated with PBMCs in 1.1% (w/v) Ultra-Pure Ca^2+^ medium viscosity high-guluronic acid (MVG) alginate (UP MVG NovaMatrix, Pronova Biomedical, Avaldsnes, Norway), prepared in 0.9% (w/v) NaCl. Cell microencapsulation in alginate was performed as described previously (61). In brief, an electrostatic-driven instrument (VARV1, Nisco, Zurich, Switzerland) was employed to obtain capsules of approximately 500 µm in diameter. The barium cation was used as cross-linking ion and polymerized capsules were maintained in their respective culture media, in 125 mL vented cap shake flasks (Corning), under orbital agitation (100 rpm). When applicable, culture media were supplemented at day 0 of culture with 100 U/mL recombinant human IL-2 (Biolegend, #589102) or 10 ng/mL recombinant human IL-15 (Biolegend, #570302).

### Antibody challenge assays

NK- and PBMC-induced cytotoxicity over SKBR3 or HCC1954 cells was assessed in 2D and 3D cultures (in direct or encapsulated co-cultures), in the culture medium of each cell line.

For 3D direct co-cultures, SKBR3 or HCC1954 aggregates (target cells) were plated directly, in ultra-low adhesion, round bottom 96-well plates (Corning, #444-1020) with NK cells or PBMCs (effector cells) at different effector (E): target (T) ratios (2:1, 5:1 and 10:1 for both effectors, 20:1 for PBMCs only). The cultures were exposed to 5 µg/mL trastuzumab (Herceptin^®^, Genentech), 10 µg/mL pertuzumab (Perjeta™, Roche), or both, for 4 days, at 37°C; the concentrations of the antibodies were defined according to literature (65, 66), stock solutions were prepared in water. Negative (cancer cells only), positive (total cancer cell death), and IgG isotype controls were also included. The latter was performed with an E (PBMCs): T ratio of 5:1 and exposed to control human IgG (67). For the positive control, used to estimate the total LDH content of the culture, cancer cell aggregates were cultured for 4 days, at 37°C and then incubated with 1% Triton-X100, for 2h, at 37°C, to induce total cell lysis. Five replicate wells were performed per condition.

For 2D assays, SKBR3 or HCC1954 cells were plated with NK cells in tissue culture treated, flat-bottom 96-well plates (Corning, #353072), at different E:T ratios (2:1, 5:1, and 10:1), in the presence of 5 µg/mL trastuzumab. As in 3D, a positive control for determination of total cell death was also included. Four replicate wells were performed per condition.

After 4 days, plates were centrifuged to pellet cells and cell debris, and the supernatants, corresponding to conditioned medium of cultures exposed to the antibodies (CM LDH_Ab_, equation 1) or conditioned medium plus cell extract of the positive control (Total LDH _control_, equation 1)) were collected. Cell death was quantified by the LDH release assay (CyQUANT™ LDH Cytotoxicity Assay, ThermoFisher, #C20301), as per manufacturer’s protocol. Cytotoxicity was calculated using the following formula (68):


Equation 1: $$Cytotoxicity\;(\%)=\;\frac{CM\;LDH\;Ab}{Mean\;\mathrm{(T}otal\;LDH\;contro\mathrm{l)}}\times 100$$

For challenges in encapsulated cultures, microencapsulated co-cultures of PBMCs with SKBR3 or HCC1954 were maintained for 4 days in their respective culture media, in 125 mL vented cap shake flasks, under orbital agitation, in medium supplemented with 10 ng/mL recombinant human IL-15. The cultures were exposed at day 0 to trastuzumab alone or to the antibody combination (trastuzumab plus pertuzumab), employing the same antibody concentrations used in 3D direct co-cultures. Cell viability was assessed by MitoView™ 633 and NucView^®^488 viability dyes and both cancer and immune cells phenotypes were characterized by flow cytometry.

### Immunofluorescence microscopy

Samples of encapsulated cultures were collected on day 4 and, when applicable, day 7. The capsules were fixed in 4% (w/v) formaldehyde with 4% (w/v) sucrose in PBS (fixation solution), for 20 min. For whole-mount immunostaining, fixed samples were permeabilized and blocked with 1% (v/v) Triton X-100 (Sigma-Aldrich) in blocking solution (0.2% (w/v) fish skin gelatin (FSG, Sigma-Aldrich) in PBS) for 3 h, at RT, followed by 2 h in blocking solution. For preparation of cryosections, fixed samples were incubated in 30% sucrose overnight at 4°C, embedded in Tissue-Tek OCT compound (Sakura Finetek USA), snap frozen in liquid nitrogen and stored at -80°C for at least two days before sectioning in a cryostat with a slice thickness of 10 µm. For immunostaining, cryosections were permeabilized with 0.1% Triton X-100 for 10 min and blocked in blocking solution for 45 min, at RT. Samples derived from 2D cell cultures were incubated for 10 min in fixation solution, permeabilized with 0.1% Triton X-100 in PBS for 10 min, followed by a 30 min incubation in blocking solution, at RT. All antibodies were prepared in blocking solution; primary antibodies were incubated overnight at 4°C (3D whole-mount and cryosections) or for 2h at RT (2D), secondary antibodies were incubated for 2h at RT. After immunolabelling, nuclei were stained with DAPI (Invitrogen, #D3571) (1:1500 in blocking solution) for 10 min, at RT. The following primary antibodies were used: anti-HER2 (1:500, rabbit polyclonal, DAKO, #A0485), anti-CK18-FITC (1:100, mouse monoclonal, Sigma-Aldrich, #F4772), anti-E-cadherin (1:100, mouse monoclonal, BD Biosciences, #610181), anti-ZO-1 (1:100, rabbit polyclonal, Thermo Fisher, #40-2200), anti-CD45-PE (1:50, mouse monoclonal, BD Pharmingen, #555483). The following secondary antibodies were used (all at 1:2000 dilution): goat anti-mouse Alexa Fluor 488 (Thermo Fisher, #A11001), goat anti-rabbit Alexa Fluor 488 (Thermo Fisher, #A11008), goat anti-mouse Alexa Fluor 594 (Thermo Fisher, #A11005), goat anti-rabbit Alexa Fluor 594 (Thermo Fisher, #A11037).

Samples from 2D monoculture were mounted with ProLong™ Gold Antifade Mountant (Invitrogen, #P36934). For 2D and 3D monocultures (3D cryosections), all images were acquired in a Zeiss LSM 880 point scanning confocal microscope with Airyscan detector; serial optical sections were acquired every 0.5 μm (2D) or 1.2 μm (3D cryosections); the Zeiss Zen 2.3 (black edition) software was used for processing of the Airyscan raw images. For imaging of encapsulated co-cultures, samples were subject to optical clearing with RapiClear 1.49 (SUNJIN Lab). Images of full capsules were acquired in a Prairie Ultima two-photon system, mounted on an Olympus BX51 body, using GaAsP/multialkali PMT detectors and GFP and RFP filtersets. Serial sections were acquired every 1 μm. Image processing, namely merging of channels and linear brightness & contrast adjustments, was performed in the ImageJ software, version 1.52i (NIH).

### Viability assessment

Cultures were sampled on days 1 and 4 and, when applicable, day 7. Samples were incubated with MitoView™ 633 and NucView^®^488 Apoptosis Assay kit (1:1000, Biotium), at 37°C, for 1h, before live imaging in a Leica DMI6000 B widefield fluorescence microscope. Image processing, namely merging of channels and linear brightness & contrast adjustments, was performed in ImageJ software, version 1.52i (NIH).

### Flow cytometry

Encapsulated cultures were incubated in a chelating solution of 10mM HEPES/100mM EDTA in MiliQ water for alginate dissolution, washed in PBS and afterwards incubated in TrypLE™ Express Enzyme (Gibco, #12604013) for dissociation of cancer cell aggregates. Samples were treated with Human TruStain FcX (Biolegend, #422302) to prevent non-specific binding of antibodies to Fc receptors and then incubated with the selected antibody cocktail in 0.2% FBS (in PBS), for 45 min, at 4°C. After immunostaining, samples were further incubated with DAPI (1:200), for 10 min, at 4° C, for assessment of cell viability. The following antibody panels were used: anti-CD45-PE (1:5, BD Pharmingen, #555483), anti-CD56-FITC (1:20, Biolegend, #318303), anti-CD16-APC (1:20, Biolegend, #360706), anti-PD-L1-BV785 (1:20, Biolegend, #329735) (*Panel 1*); anti-CD45-APC-R700 (1:20, BD Pharmingen, #566042), anti-CD3-KIRAVIABlue520 (1:20, Biolegend, #300482), anti-CD56-PE/Dazzle594 (1:20, Biolegend, #318348), anti-CD14-PE (1:20, Biolegend, #301806) anti-CD16-APC, anti-PD-L1-BV785 (*Panel 2*). Samples were analyzed by flow cytometry on the BD FACSCelesta™ Cell Analyzer (BD Biosciences) and data analysis was performed using FlowJo™ v10.7.2 software (BD Life Sciences). A flowchart of the gating strategy for each antibody panel is presented in **Supplementary Figure 2A**.

### Cytokine antibody arrays

Supernatants from encapsulated co-cultures were collected on day 4 and stored at -80°C until use. The Human Cytokine Antibody Array (42 targets) (Abcam, #ab133997), performed according to the manufacturer’s protocol, was used to detect cytokine expression in culture supernatants after centrifugation at 14000 RPM for 10min to remove cell debris. Detection of the chemiluminescent signal was performed in the iBright™ FL1500 imaging system (Thermo Fisher), with exposure times ranging from 0.5sec to 10sec. Signal quantification was performed in ImageJ software (NIH), with the highest exposure time for which there was no saturation, by applying same-area Region-Of-Interest to each individual antibody spot in each array membrane and measuring signal intensity. Semiquantitative assessment of cytokine expression was obtained by: 1) subtracting background signal (average of blank spots) to all spots of each membrane; 2) filtering out measurements with a value equal or inferior to: avg(blank) + 2*SD(blank); 3) normalizing all spots of each membrane to the same reference membrane (Equation 2); 4) normalizing each spot to the membrane’s positive control; 5) calculating the average of technical duplicates; 6) calculating fold change between experimental conditions (dual antibody challenge *vs* no antibody).


Equation 2: $$X_{n}\left(y\right)=X\left(y\right)*\frac{P(ref)}{P\left(y\right)}$$

Where:

X_n_(y) = normalized signal for spot “X” on membrane “y”

X(y) = signal for spot “X” on membrane “y”

P(ref) = average signal of positive control spots on reference membrane

P(y) = average signal of positive control spots on membrane “y”

### Statistics

All depicted experiments result from at least three independent biological replicates, except those depicted in **Supplementary Figure 3D and E** (n = 2). In the case of experiments involving PBMCs or NK cells, each biological replicate was performed with immune cells from a different healthy donor. Plots were created and statistical analysis was performed using Graphpad Prism version 9.0.0. Comparison of multiple groups was performed with one-way ANOVA test and comparison between two groups was performed with t-test; specific flow cytometry analyses of encapsulated co-cultures were performed with paired t-test, pairing observations from the same PBMC donor. p-values inferior to 0.05 were considered significant. Statistical significance denoted in the following manner: one symbol, p<0.05; two symbols, p<0.01; three symbols, p<0.001. Error bars display standard deviation. Further statistical details can be found in each figure legend.

## Results

### HER2-OE BC cells maintain identity features in encapsulated spheroid cultures

**Figure 1:**
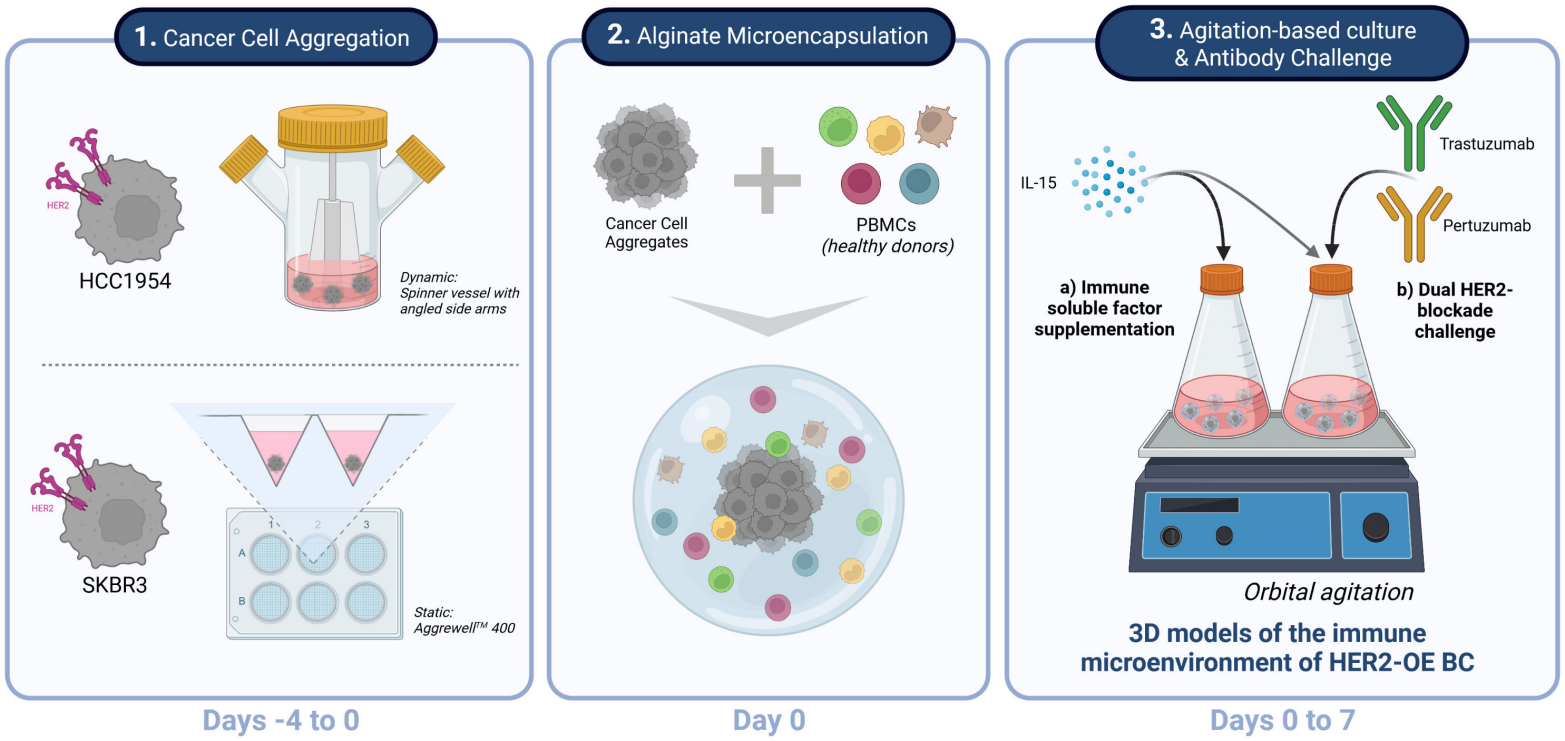
Experimental strategy for generation, culture and challenge of HER2^+^ breast cancer 3D models of the immune microenvironment of human breast cancer overexpressing the epidermal growth factor receptor 2 (HER2-OE BC). Schematic representation of the three stages of the process: 1 - The HER2-overexpressing breast cancer cell lines HCC1954 and SKBR3 were aggregated in dynamic and static conditions, respectively, to assure formation of stable 3D structures independently of the intrinsic cell-cell adhesion properties of each cell line (Days -4 to 0). 2 - Co-cultures of the HER2-OE breast cancer cell lines and peripheral blood mononuclear cells (PBMCs) derived from healthy donors were established by alginate microencapsulation (Day 0); 3 – Encapsulated co-cultures were supplemented with IL-15 and kept under orbital agitation, for up to 7 days. A proof-of-concept of a therapeutic challenge was performed with a combination of two anti-HER2 therapeutic antibodies currently employed in the clinics, trastuzumab and pertuzumab. Created with BioRender.com.

**Figure 2:**
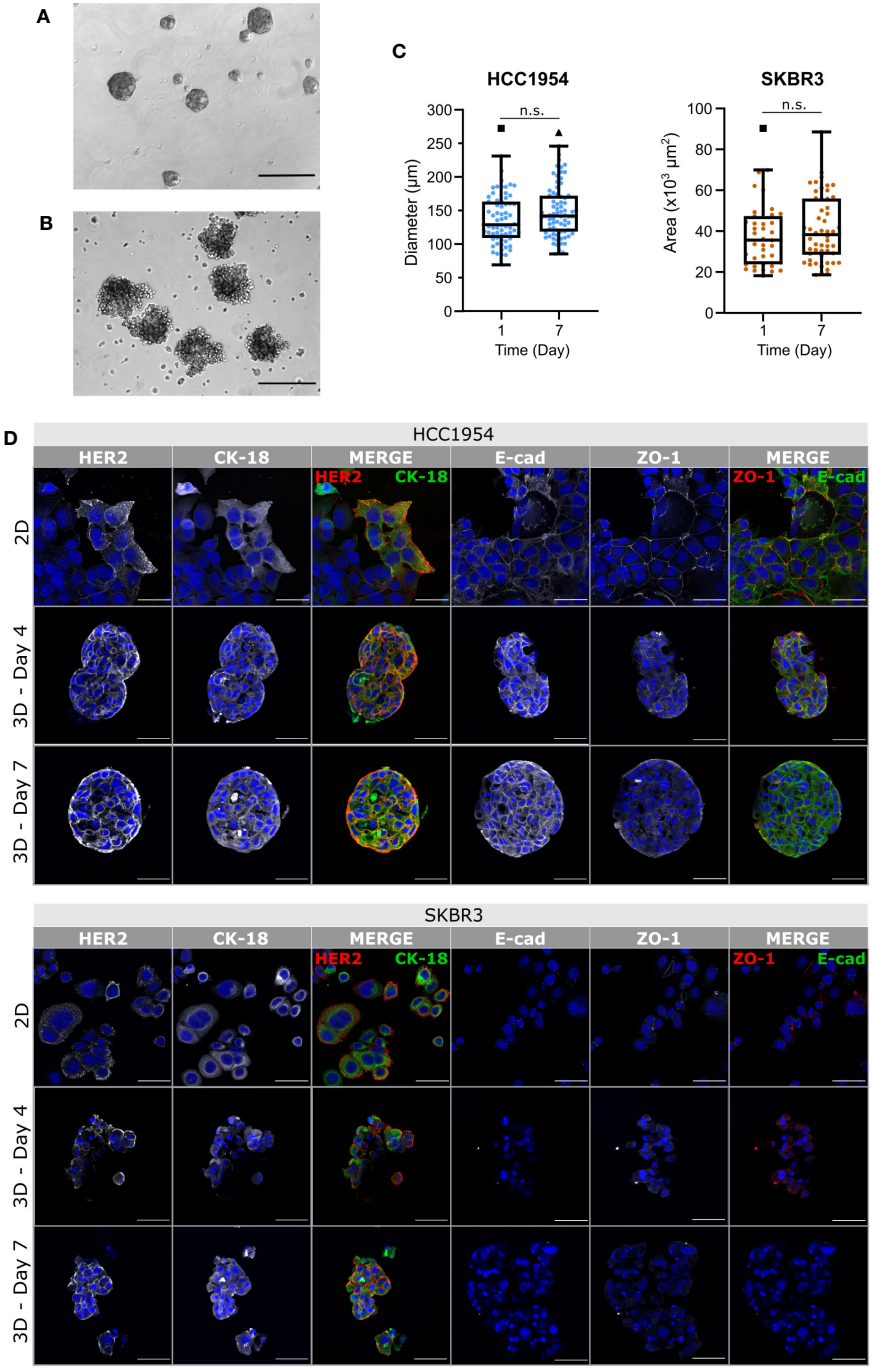
Phenotypic traits of HCC1954 and SKBR3 cells were retained in agitation-based encapsulated aggregate cultures. **(A, B)** Phase contrast microscopy images of HCC1954 **(A)** and SKBR3 **(B)** aggregates, immediately before encapsulation (Day 0), evidencing the different morphology and compactness of aggregates of each cell line. Scale bars: 300 µm. **(C)** Size of HCC1954 and SKBR3 aggregates, evaluated by diameter or area, respectively. Throughout the seven days of both encapsulated cultures, there were no significant changes in aggregate size. Box and whiskers plots represent pooled measurements from optical microscopy images of at least 3 independent experiments (HCC1954: ≥16 aggregates per experiment, 66 to 76 total aggregates per condition; SKBR3: ≥8 aggregates per experiment, 39 to 52 total aggregates per condition). Pairwise statistical comparisons between indicated groups performed with unpaired t-test: n.s., p>0.05. **(D)** Immunodetection of breast cancer identity markers in 2D monolayers and encapsulated aggregates of the HCC1954 (upper panel) and SKBR3 (lower panel) cell lines. Detection of human epidermal growth factor receptor 2 (HER2, red) and cytokeratin-18 (CK-18, green); zonula occludens 1 (ZO-1, red) and E-cadherin (E-cad, green) suggested preservation of HER2-overexpressing breast cancer phenotype, for at least 7 days of culture. Nuclei were stained with DAPI (blue). Each image of the encapsulated 3D monocultures displays a maximum intensity projection of two 1.2 µm consecutive optical slices of a cryosection. Scale bars: 50 µm. Representative pictures depicting one of 3 independent experiments.

With the goal of generating an *in vitro* model of HER2-OE BC immune microenvironment, we adopted a co-culture strategy previously described by our group (60, 61). The method is based on microencapsulation in alginate of tumor cell spheroids and distinct TME cell types, and agitation-based culture systems. As summarized in **Figure 1**, after a first step of cancer cell aggregation, cancer cell aggregates and PBMCs are co-encapsulated in an alginate hydrogel. Finally, the heterotypic cell cultures are attained by maintaining the microcapsules under orbital agitation, for at least 4 days, to promote tumor-immune cell interactions. To represent the HER2-OE BC surrogate subtype, we used HCC1954 and SKBR3, two cell lines positive for HER2 and negative for the estrogen and progesterone receptors (58, 69). The aggregation strategy differed for each of the cell lines due to their reported distinct cell-cell adhesion properties (63, 70–75), and the distinct morphologies in 3D (63, 71). HCC1954 aggregates were generated in spinner vessels, forming compact spheroid structures (**Figure 2A**), as previously described (63, 64), with an average diameter of 138 (± 39) µm (**Figure 2C**). For SKBR3 cells, known to form grape-like loose structures with poor cell-cell adhesion (71–75), aggregates were generated in Aggrewell™ 400 plates. Despite their irregular shape (**Figure 2B**), these aggregates displayed sufficient cohesion to allow manipulation and further microencapsulation, suggesting an improvement over other matrix-free aggregation strategies (73–75).

Aggregates of both HCC1954 and SKBR3 cell lines microencapsulated in alginate, and maintained under agitation, conserved their original shape (**Supplementary Figure 1A**) and size (**Figure 2C**) throughout the culture period (up to seven days). Encapsulated tumor monocultures maintained high cell viability during the first four days of culture, with few apoptotic cells detected in the aggregates. On day 7, there was an increase in apoptotic cells detected compared with day 4. However, these cells were still few, individualized and distributed equally throughout the aggregates (**Supplementary Figure 1A**). The presence of a few apoptotic cells dispersed throughout the SKBR3 aggregates was also reported elsewhere (74).

Immunofluorescence detection of BC cell markers showed that the plasma membrane localization of the HER2 receptor, typically detected in 2D cultures of both cell lines (75–77) (**Figure 2D**, upper rows, first column) was retained in the 3D cultures throughout the seven days of culture (**Figure 2D**, middle and lower rows, first column). Cytokeratin-18 (CK-18), an intermediate filament expressed in epithelial tumor cells, including breast (78–80), was detected in both cell lines in 2D and in 3D encapsulated culture (**Figure 2D**, second column). E-cadherin was detected in HCC1954 cells, both in 2D and in 3D, localizing mostly to the plasma membrane (**Figure 2D**, fourth column), but not in SKBR3 cells, in line with the reported *CDH1* deletion in this cell line (81, 82). The tight junction protein Zonula occludens-1 (ZO-1) was also detected at the plasma membrane of HCC1954 cells, in 2D and 3D cultures; lower levels of ZO-1 were detected in 2D cultures of SKBR3 cells (**Figure 2D**, fifth column), as reported for this cell line (70, 83). ZO-1 membrane detection decreased in SKBR3 3D cultures, consistent with the poor cell-cell contact described for this cell line under these conditions (71). These observations indicate that the expression of BC markers in encapsulated 3D aggregates is mostly similar to the well characterized 2D cultures and to previous reports for cell aggregates and show they can be maintained for up to one week in culture, supporting their use for the development of *in vitro* models of the HER2-OE BC microenvironment.

### 3D models of HER2-OE BC immune microenvironment retain the NK cell population

**Figure 3:**
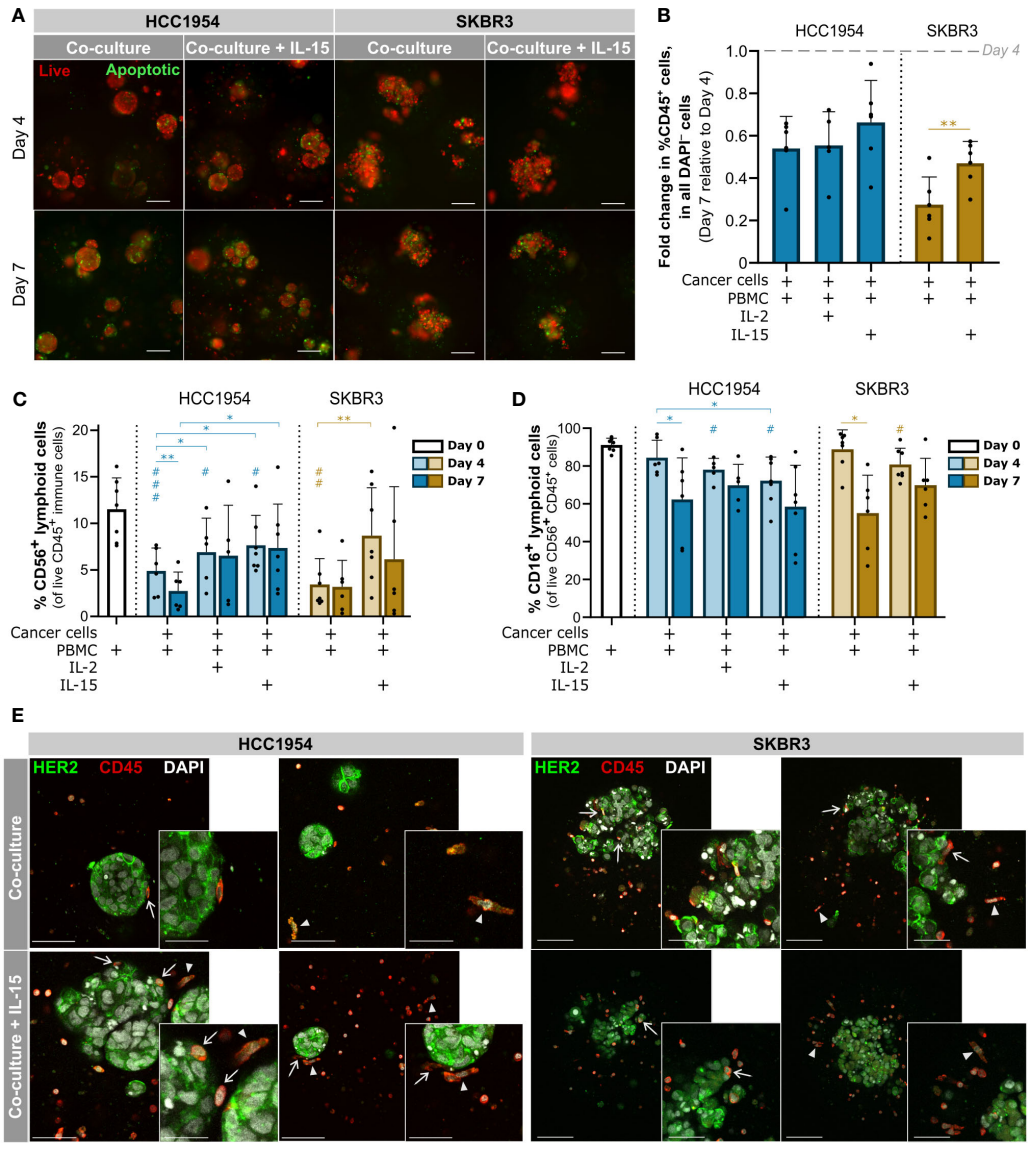
3D models of the immune microenvironment of HER2-OE BC retained the immune cell compartment, including NK cells, for at least 7 days. **(A)** Viability assessment with the fluorescent probes Mitoview (live cells, red) and Nucview (apoptotic cells, green) showed an increase in apoptotic cells from day 4 to day 7, scattered throughout the aggregates of HCC1954 (left) and SKBR3 (right) co-cultured with PBMCs, regardless of IL-15 supplementation. Representative pictures depict one of 6 independent experiments, performed with different immune donors. Scale bars: 150 µm. Full panel for 3 timepoints and all culture conditions in **Supplementary Figure 1**. **(B)** Fold change in the percentage of immune cells (CD45^+^) within all viable cells (DAPI^−^), from day 4 to day 7 of culture, detected by flow cytometry; there was a sharp decrease in the proportion of the immune compartment, in both HCC1954 (blue) and SKBR3 (yellow), which was partially rescued in SKBR3 co-cultures supplemented with IL-15. **(C)** Percentage of CD56^+^ lymphoid cells, including NK cells within the viable immune cell population (CD45^+^ DAPI^−^), detected by flow cytometry. IL-15 supplementation had a positive effect in the NK cell population, in co-cultures with HCC1954 and SKBR3 cell lines. **(D)** Percentage of CD16^+^ CD56^+^ lymphoid cells within the viable CD56^+^ lymphoid cell population (CD56^+^ CD45^+^ DAPI^−^), by flow cytometry. Despite the small reduction of CD16^+^ cells induced by 4-day cytokine stimulation in both co-cultures with HCC1954 and SKBR3, by day 7 the percentages were similar to non-supplemented conditions. **(B–D)** Cell populations were identified and quantified according to gating strategy depicted in **Supplementary Figure 2A** (Panel 1). Day 0 corresponds to the PBMC population immediately before encapsulation. Bars represent mean ± S.D. from N = 6 independent experiments, performed with different immune donors. Pairwise statistical comparisons between indicated groups (*) or relative to day 0 (#), performed with paired t-test; *,# p<0.05; **,##, p<0.01, ###, p<0.001. **(E)** Immunodetection of epidermal growth factor receptor 2 (HER2, green) and CD45 (red), in co-cultures of HCC1954 or SKBR3 with PBMCs (left and right panels, respectively), without or with IL-15 supplementation (upper and lower row, respectively), by 2-photon emission fluorescence microscopy. At day 4, of CD45^+^ immune cells had an elongated morphology (arrowheads) and established direct contact with HCC1954 and SKBR3 HER2-OE breast cancer cells (arrows). Each image displays a maximum intensity projection of two consecutive optical slices of 1 µm within an entire capsule. Representative pictures depict one of 3 independent experiments, performed with different immune donors. Scale bars: 150 µm (main), 75 µm (inset).

To establish heterotypic 3D cell cultures, aggregates from both HER2^+^ cell lines were microencapsulated with PBMCs obtained from different human donors. In doing so, we aimed to reflect in these models the natural variability of immune phenotypes and responses found in the human population (84) and depict molecular interactions between immune populations found in the tumor microenvironment (e.g., lymphocytes, monocytes/macrophages, NK cells). We used a non-functionalized alginate hydrogel for immobilization of cells in microcapsules of approximately 500 µm diameter (mean = 476.6 ± 38 µm). Microencapsulation not only promoted proximity between the tumor and immune cellular compartments but also allowed the use of an agitation-based culture system for improved and homogeneous mass transfer compared to static cultures, and culture monitoring by non-destructive sampling (60, 61). Encapsulated co-cultures of HER2^+^ BC aggregates and PBMCs were maintained for seven days and monitored for cell viability and immune cell phenotype. During the first four days, co-cultures of both BC cell lines displayed high viability without the formation of necrotic cores, with low and scattered detection of apoptotic cells (**Figure 3A**), and the aggregate shape was maintained. At day 7, apoptosis detected by caspase 3 activity was slightly higher than at day 1. Apoptotic cells were distributed throughout the aggregates (**Supplementary Figure 1A**), especially in the HCC1954 co-cultures, similar to what was observed in monocultures. These results suggested that immune cells are not sufficient to induce tumor clearance.

Regarding the immune compartment, from day 4 to day 7 there was a significant loss of viability of the immune cells in co-cultures (**Supplementary Figure 1C**, **, p<0.01, paired t-test). At day 7 of co-culture, the live immune cell compartment decreased to approximately 54% and 28% (in HCC1954 and SKBR3 co-cultures, respectively) of those observed at day 4 (**Figure 3B**). NK cells are considered the main effectors of the immune mechanism of action of trastuzumab, quickly inducing ADCC after target recognition (85). For this reason, we identified and characterized the NK cell population in co-cultures. We employed a gating strategy on live CD45^+^ cells based on the selection of small, low complexity lymphoid cells expressing the surface receptor CD56 (CD56^+^), either at low (CD56^dim^) or high (CD56^hi^) levels (**Supplementary Figure 2A**, “Panel 1”). In both co-cultures, the representation of the CD56^+^ lymphoid cell population in the immune compartment was significantly reduced from day 0 (PBMCs after isolation) to day 4 of culture (**Figure 3C**, ##, p<0.01, paired t-test). Supplementation with IL-15, commonly used to improve activation of NK cells *in vitro* (85, 86), partially rescued the CD56^+^ lymphoid cell fraction in HCC1954 co-cultures (**Figure 3C**, *, p<0.05, paired t-test). For SKBR3 co-cultures, IL-15 supplementation sustained the proportion of the CD56^+^ lymphoid cell compartment with no significant difference to the original PBMC population (**Figure 3C**, paired t-test). These data evidenced the role of cytokine supplementation in the preservation of the NK immune subset. Moreover, the proportion of the immune compartment in SKBR3 co-cultures, but not HCC1954, was also partially rescued by IL-15 supplementation at day 7 (**Figure 3B**). We have also tested IL-2 supplementation in HCC1954 co-cultures, as this cytokine is reported to improve NK cell viability (9, 85), but the results were not superior to those obtained with IL-15 in what concerns CD56^+^ lymphoid cells and the global immune compartment (**Figure 3B, C** and **Supplementary Figure 1C**).

The CD16 receptor is responsible for recognizing and binding to the Fc portion of IgG antibodies such as trastuzumab, thus initiating ADCC against the opsonized tumor cell (6, 85). Therefore, we assessed the CD16^+^ NK cell subset in the different co-culture conditions, along culture time (**Figure 3D**). At day 4 of co-culture of HCC1954 and SKBR3 supplemented with IL-15, we observed a small reduction of approximately 19% and 11%, respectively, in the CD16^+^ subset of the CD56^+^ CD45^+^ lymphoid cell population when compared to the original PBMC fraction (**Figure 3D**, #, p<0.5, paired t-test). In co-cultures without cytokine supplementation, there was no significant reduction in the percentage of CD16^+^ CD56^+^ lymphoid cells from day 0 to day 4 (**Figure 3D**). The reduction in CD16^+^ detection upon 4 days of IL-15 supplementation suggested a cytokine-mediated activation of NK cells, which has been associated with proteolytic downregulation of plasma membrane CD16 by ADAM-17 (87); the latter was shown to be activated by IL-15 in combination with other TME-associated cytokines (88). On the other hand, at day 7 the CD16^+^ CD56^+^ lymphoid cell subset was stable across supplementation conditions and significantly reduced in co-cultures without cytokine supplementation, when compared with day 4 (**Figure 3D**, *, p<0.5, paired t-test). In summary, the proportion of NK cells in culture was higher with cytokine supplementation (**Figure 3D**), with no significant differences in the representation of the CD16^+^ NK subset obtained at day 7 of co-cultures with and without cytokines.

We assessed the cellular distribution and spheroid morphology within co-cultures employing 2-photon excitation fluorescence (2PEF) microscopy. On day 4, CD45^+^ immune cells were dispersed throughout the alginate capsules, varying in morphology from small round-shaped leukocytes to more extended phenotype (**Figure 3E**, arrowheads in both panels). We detected few apparent direct contacts between immune cells and HCC1954 cells at the surface of the aggregates but not infiltration, both in the co-cultures without and with IL-15 supplementation (**Figure 3E**, left panel, arrows). On the other hand, in SKBR3 co-cultures, we observed multiple close contacts between the two cell types, with CD45^+^ cells located not only around the cancer aggregates but also in inner regions, in between cancer cells (**Figure 3E**, right panel, arrows), which is consistent with the reduced compactness of these aggregates compared with HCC1954. In co-cultures of both cancer cell lines, aggregates retained the morphology and the HER2 localization at the cell surface, as observed in monocultures (**Figures 3E** and 2D, respectively). Few apoptotic cells were identified by chromatin condensation (**Figure 3E**), corroborating the caspase activity detection results (**Figure 3A**).

Overall, at day 4 of co-culture with IL-15 supplementation, high cell viability and a better representation of the immune cell compartment was attained, including of the NK cell population, while still maintaining elevated levels of CD16. Therefore, we set these culture conditions and time-point to assess the immunomodulatory functions of the anti-HER2 antibody challenge.

### 3D models of the HER2-OE BC immune microenvironment are susceptible to dual HER2 blockade, which promotes NK cell activation

**Figure 4:**
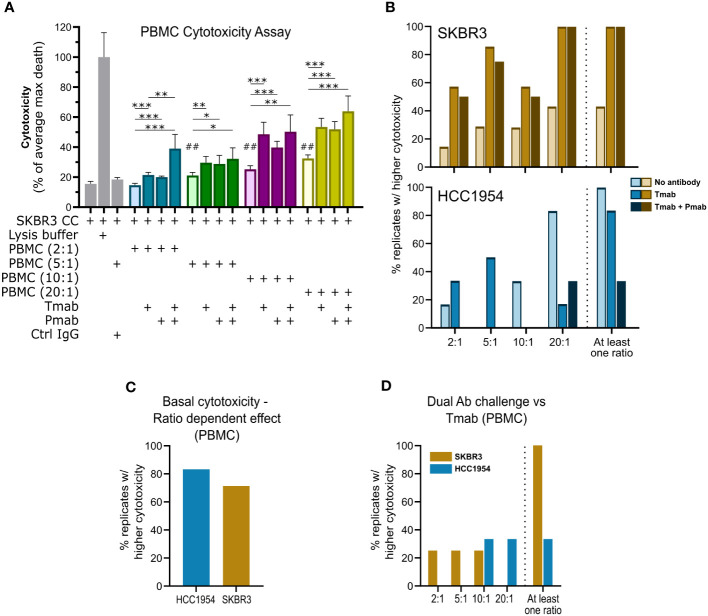
Single and dual anti-HER2 antibody challenge can induce ADCC against aggregates of HER2-OE breast cancer cell lines via PBMC. **(A)** Direct cytotoxicity assay performed with SKBR3 aggregates and PBMCs at different effector (PBMC): target (cancer cell) ratios (E:T; 2:1, blue; 5:1, green; 10:1, purple; 20:1, yellow), by assessment of LDH leakage after 4 days of challenge. Tested conditions included negative (cancer cells only) control; positive (cancer cells with lysis buffer) control, to determine maximal cancer cell death (max death); IgG isotype control (Ctrl IgG), assayed at 5:1; no challenge, single (trastuzumab, Tmab) or dual (trastuzumab plus pertuzumab, Tmab + Pmab) antibody challenge, assayed at the different E:T. Data from one experiment representative of those depicted in B; bars represent mean ± S.D. of five technical replicates. Pairwise statistical comparisons between indicated groups (*) or between the indicated condition and negative control (#) performed with unpaired t-test: *, p<0.05; **, ##, p<0.01, ***, p<0.001. **(B)** Assessment of the percentage of biological replicates displaying increased cytotoxicity (cell death measured by LDH leakage) in the 4-day antibody challenge in direct co-cultures with PBMCs and SKBR3 (yellow/brown, above) or HCC1954 (blue, below) cancer cell aggregates, at different E:T ratios, demonstrated that PBMC basal cytotoxic ability (light shade) could be boosted by Tmab (medium shade) and by combination with Pmab (dark shade). Plot indicates percentage of biological replicates for which tumor cell death was significantly (p<0.05) higher: in the presence of PBMC than in negative control with only cancer cells (no antibody); in the presence of PBMC + Tmab than in the presence of PBMCs only (Tmab); or in presence of PBMC + Tmab + Pmab than in the presence of PBMCs only (Tmab + Pmab). The percentage of biological replicates displaying increased cytotoxicity in at least one of the ratios tested (At least one ratio) is also plotted. PBMCs from 7 (Tmab challenge) or 4 (Tmab + Pmab challenge) distinct donors were assayed with SKBR3 aggregates; PBMCs from 6 (Tmab) or 3 (Tmab + Pmab) different donors were assayed) with HCC1954 aggregates. Assays for each donor were performed independently. **(C)** Assessment of the percentage of biological replicates displaying increased SKBR3 (yellow) or HCC1954 (blue) cell death (measured by LDH leakage after 4 days of co-culture with PBMCs), with increasing E:T ratios, revealed that the basal cytotoxic function (without antibody challenge) of PBMC against HER2^+^ breast cancer cells is dose-dependent. Plot indicates percentage of biological replicates in which the trend of increased tumor cell death with increased E:T ratios was significant (p<0.05), as evaluated by one-way ANOVA, for the four E:T ratios, for each of the cancer cell lines. PBMCs from 7 (SKBR3) or 6 (HCC1954) different donors, assayed independently. **(D)** Assessment of the percentage of biological replicates in which SKBR3 (yellow) or HCC1954 (blue) cell death (measured by LDH leakage after 4 days of challenge in co-culture with PBMCs) was significantly (p<0.05) higher in the presence of Tmab + Pmab than Tmab, at the different E:T ratios, and the percentage of biological replicates in which a higher effect of the dual HER2 blockade was observed in at least one of the ratios tested (“At least one ratio”), pointed to a superiority of the dual antibody challenge over the single-agent trastuzumab in enhancing immune-mediated tumor cell killing. Plot indicates percentage of biological replicates in which tumor cell death was significantly (p<0.05) higher in dual blockade that in Tmab alone, evaluated by unpaired t-test for each of the E:T ratios, for each of the cell lines. PBMCs from 4 (SKBR3) or 3 (HCC1954) different donors, performed independently.

We performed a proof-of-concept study to demonstrate the ability of the human PBMC-derived immune cells to elicit antibody-dependent cell-mediated cytotoxicity (ADCC) in the model, i.e., to display cytotoxic function against the HER2^+^ cell lines in aggregates, in presence of the anti-HER2 therapeutic antibodies. We co-cultured cancer aggregates and immune cells in 96-well plates, at different effector-to-target (E:T) ratios. The direct co-cultures were challenged with trastuzumab, pertuzumab or the antibody combination and, after 4 days, cell death was assessed by lactate dehydrogenase (LDH) release (**Figure 4A**). In PBMC cytotoxicity assays, primary immune cells demonstrated a native ability to induce cancer cell death (basal cytotoxicity) for both cell lines. Cell death was significantly increased in the presence of PBMCs compared with the negative control (cancer cells only, **Figure 4B**), with most donors displaying an increase in basal cytotoxicity at higher E:T ratios (**Figure 4C**). In this assay, SKBR3 aggregates were the most susceptible to ADCC, induced by trastuzumab alone or in combination with pertuzumab, in accordance with previous reports in 3D (89–91). We observed a significant increase in cell death in all E:T ratios tested, compared with the respective ratio without antibody challenge (**Figure 4B**), whereas incubation with a non-specific, isotype-matched IgG control antibody (67) had no effect on immune-mediated cell death (**Figure 4A**). These data showed that the immune response was triggered specifically by the binding of trastuzumab and pertuzumab to the HER2 receptor. It is also worth noting that, although cells from only 40% of the PBMC donors displayed basal cytotoxicity against SKBR3 aggregates, cells from all donors displayed ADCC in at least one of the ratios tested. These results highlight the suitability of this cell line to study the contribution of anti-HER2 therapeutic antibodies for anti-tumor immune function. The HCC1954 cell line was less susceptible to ADCC in our assay conditions, similar to what was reported in other assay configurations (89–91). Immune cells from only 80% of the PBMC donors displayed increased cytotoxic function in presence of the anti-HER2 antibodies; nonetheless, all donors had the native capacity to induce cell death in at least one of the ratios tested (**Figure 4B**). Interestingly, the ability of the trastuzumab and pertuzumab combination to induce ADCC over SKBR3 cells was significantly higher than that of trastuzumab alone, for all donors tested (**Figure 4D**) (92, 93). This effect was also observed for HCC1954, but in a donor-dependent manner (**Figure 4D**). As previously reported (65, 66, 92, 93), this cytotoxic effect appears to be at least partly mediated by NK cells, as parallel cytotoxicity assays with NK cells isolated from PBMCs displayed enhanced cytotoxic activity in presence of trastuzumab compared to the co-culture without antibody challenge (**Supplementary Figures 3A and C**). The lower susceptibility of HCC1954 cells to ADCC was also observed in a 2D cytotoxicity assay with PBMC-derived NK cells (**Supplementary Figures 3D and E**) (89, 90). We further verified that the spontaneous and maximum death of the immune cells was negligible in this experimental setup (**Supplementary Figure 3B**), supporting that LDH leakage was derived from cancer cell death.

**Figure 5:**
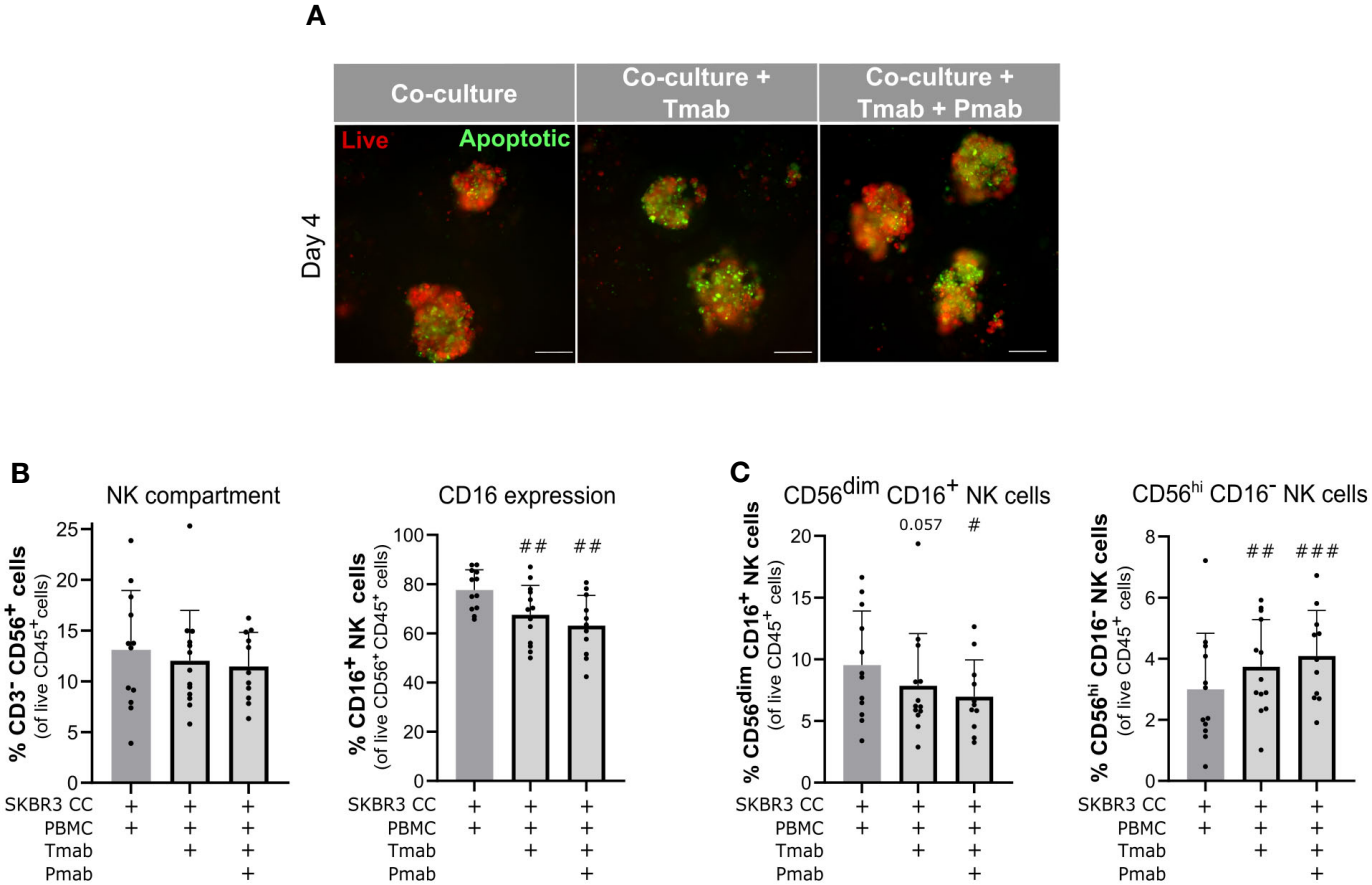
Challenge of SKBR3-based 3D models of the immune microenvironment of HER2-OE BC with anti-HER2 antibodies induced cancer cell apoptosis and downmodulation of CD16 in NK cells. **(A)** Viability assessment with the fluorescent probes Mitoview (live cells, red) and Nucview (apoptotic cells, green) revealed an increase in apoptosis in SKBR3 aggregates upon 4 days of single (trastuzumab, Tmab) or dual (trastuzumab plus pertuzumab, Tmab + Pmab) anti- epidermal growth factor receptor 2 (HER2) antibody challenge. Co-cultures were supplemented with IL-15. Representative pictures depict one of 11 independent experiments, performed with different immune donors. Scale bars: 150 µm. Full panel for 2 timepoints and all culture conditions in **Supplementary Figure 3**. **(B)** Percentage of NK cells (CD3^−^CD56^+^) and CD16^+^ NK cells (CD3^−^CD56^+^CD16^+^) within the viable immune cell population (CD45^+^ DAPI^−^), detected by flow cytometry. NK cells representativeness was not impacted by the 4-day single and dual anti-HER2 antibody challenges but the CD16^+^ NK subset was partially depleted. Co-cultures were supplemented with IL-15. **(C)** Percentage of the two major NK cell subsets (CD3^−^CD56^dim^CD16^+^ and CD3^−^CD56^+^CD16^–^) in the viable immune cell population (CD45^+^DAPI^−^), detected by flow cytometry. Both NK cell subsets were modulated upon the 4-day single and dual anti-HER2 antibody challenges. Co-cultures were supplemented with IL-15. **(B, C)** Cell populations were identified and quantified according to gating strategy supplied in **Supplementary Figure 2A** (Panel 2). Bars represent mean ± S.D. of N = 11 independent experiments, performed with different immune donors. Pairwise statistical comparisons, relative to co-cultures with IL-15 and without antibody challenge, evaluated by a paired t-test: #, p<0.05, ##, p<0.01, ###, p<0.001.

Having confirmed PBMC function against aggregates of the two BC cell lines used, in the presence of anti-HER2 antibodies, we challenged the encapsulated 3D co-cultures with the antibody combination (trastuzumab plus pertuzumab) or trastuzumab alone and compared cancer and immune cell phenotype with untreated control. After four days of antibody challenge, apoptotic cells were abundantly detected in co-cultures of SKBR3 (**Figure 5A**) and HCC1954 (**Supplementary Figure 4A**, bottom), while in earlier culture time-points and in controls without antibody challenge rare apoptotic cells were observed (**Supplementary Figure 4A**). Given the well-established role of NK cells in the anti-tumor activity of trastuzumab (62, 85), also observed in the direct co-culture assays (**Supplementary Figure 3A**), we assessed the modulation of the NK cell phenotype induced by anti-HER2 antibody challenge in the models. While the proportion of the NK cell population (CD45^+^ CD3^−^ CD56^+^) was unaltered in the co-cultures of the two cancer cell lines (**Figure 5B** and **Supplementary Figure 4B**), both single and dual HER2 blockade led to a significant reduction in the CD16^+^ NK cell population in SKBR3 co-cultures (**Figure 5B**). We also observed a modulation of NK cell subset representation, with a significant increase in the proportion of CD56^hi^ CD16^−^ NK cells and decrease in the proportion of the CD56^dim^ CD16^+^ NK subset (**Figure 5C** and **Supplementary Figure 2B**). This modulation is in agreement with what has been described for trastuzumab-induced ADCC (6, 87, 94). In the HCC1954 co-cultures (**Supplementary Figures 4B and C**), no modulation of NK cell subsets was detected, once again pointing to the existence of cell line-specific differences in the interaction of cancer cells with the immune compartment.

### Dual HER2 blockade induces PD-L1 downmodulation and reshapes the immune compartment of HER2-OE BC

**Figure 6:**
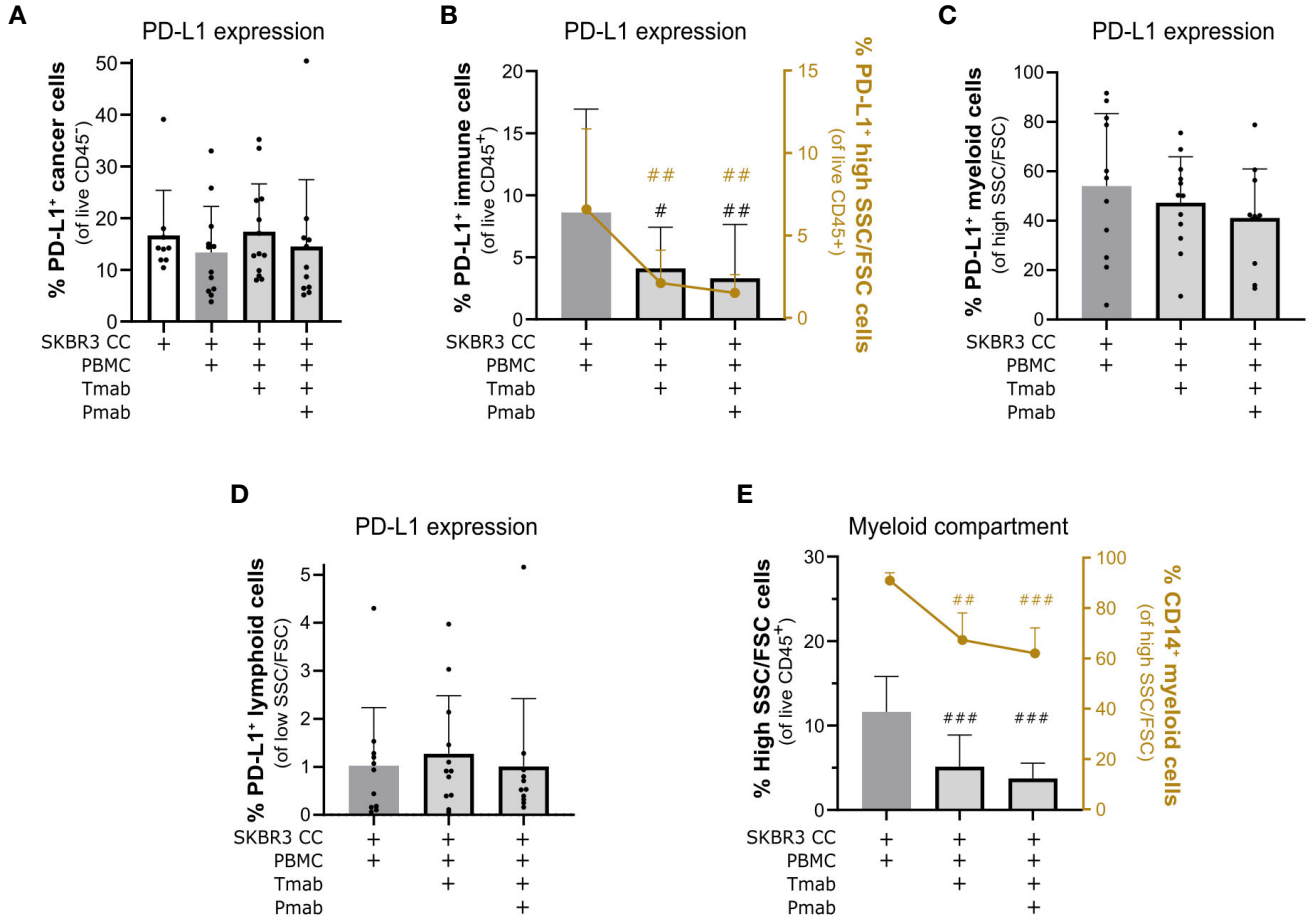
Challenge of SKBR3-based 3D models of the immune microenvironment of HER2-OE BC with anti-HER2 antibodies led to downmodulation of PD-L1 in immune cells and a partial depletion of the myeloid compartment. **(A)** Percentage of programmed death-ligand 1 (PD-L1)^+^ cancer cells within all viable cancer cells (CD45^−^ DAPI^−^), detected by flow cytometry. Detection of the PD-L1 checkpoint ligand in cancer cells was not significantly affected by the 4-day single (trastuzumab, Tmab) and dual (trastuzumab plus pertuzumab, Tmab + Pmab) anti-epidermal growth factor receptor 2 (HER2) antibody blockade. Co-cultures were supplemented with IL-15. **(B)** Percentage of PD-L1^+^ immune cells within all viable immune cells (CD45^−^ DAPI^−^, left Y axis, black) and the percentage of PD-L1^+^ live myeloid cells (PD-L1^+^SSC^hi^/FSC^hi^ in the total CD45^+^ DAPI^−^ viable immune cell population, right Y axis, yellow), detected by flow cytometry. The 4-day single and dual anti-HER2 antibody challenge induced a reduction of PD-L1 in the global immune population and, specifically, in the myeloid compartment. Co-cultures were supplemented with IL-15. **(C)** Percentage of PD-L1^+^ live myeloid cells (PD-L1^+^ in the total SSC^hi^/FSC^hi^ CD45^+^ DAPI^−^ live myeloid cells), detected by flow cytometry. The tendency to downregulate PD-L1 expression in the myeloid population upon 4 days of single and dual anti-HER2 antibody challenge was not significant. **(D)** Percentage of PD-L1^+^ live lymphoid cells (PD-L1^+^ in the total SSC^low^/FSC^low^ CD45^+^ DAPI^−^ live lymphoid cells), detected by flow cytometry. Detection of PD-L1 in lymphoid cells was not significantly affected by the 4-day single and dual anti-HER2 antibody challenge. **(E)** Percentage of live myeloid cells (SSC^hi^/FSC^hi^ in the CD45^+^ DAPI^−^ total viable immune cell population, left Y axis, black) and percentage of CD14^+^ monocytic cells (CD14^+^ in the total SSC^hi^/FSC^hi^ CD45^+^ DAPI^−^ live myeloid cells, right Y axis, yellow), detected by flow cytometry. The 4-day single and dual anti-HER2 antibody challenge induced a decrease in the proportion of myeloid cells within the immune compartment, particularly the CD14^+^ monocytic cells. Co-cultures supplemented with IL-15. **(A–E)** Cell populations were identified and quantified according to gating strategy supplied in **Supplementary Figure 2A** (Panel 2). Bars represent mean ± S.D. of N = 6 (quantification of CD14^+^ SSC^hi^/FSC^hi^ CD45^+^ DAPI^−^) and N = 11 (all other quantifications) independent experiments, performed with different immune donors. Pairwise statistical comparisons, relative to co-culture with IL-15 and without antibody challenge, evaluated with a paired t-test: #, p<0.05, ##, p<0.01, ###, p<0.001. **(B, E)** plots displaying individual data points are provided in **Supplementary Figure 6**.

To date, the few studies exploring the dynamics of the tumor microenvironment of HER2^+^ BC upon trastuzumab treatment have shown that application of this anti-HER2 antibody can impact the immunosuppressive features of the TME, namely by modulating expression of checkpoint ligand PD-L1 both in cancer and immune cells (28, 29, 32, 91, 95). In our system, we observed a significant decrease in PD-L1 expression in HCC1954 cancer cells at day 4, induced solely by the co-culture with PBMC (**Supplementary Figure 5A**), but no modulation in cancer cells upon antibody challenge, in either of the models (**Figure 6A** and **Supplementary Figure 5A**). PD-L1 modulation may occur early in the establishment of the tumor microenvironment of HER-OE BC, which may justify the discrepancy between our results and previous reports (91). Interestingly, trastuzumab challenge of SKBR3 co-cultures, both with and without pertuzumab, led to a significant downregulation of PD-L1 in immune cells (**Figure 6B**). Similarly, PD-L1 expression in immune cells of HCC1954 co-cultures was also significantly downregulated but only with the dual antibody challenge (**Supplementary Figure 5B**, left plot, left Y axis), suggesting that this model is not refractory to the immuno-modulative effects of anti-HER2 antibodies but may display a higher threshold of response. To better understand which immune populations were affected, we assessed PD-L1 expression in the lymphoid (low size and complexity) and myeloid (high size and complexity) immune compartments (**Supplementary Figure 2A**). In both co-cultures, we observed a significant drop in the proportion of PD-L1-expressing myeloid cells, within the PBMC population, of 69.5 ( ± 25.6) % (SKBR3) and 39.2 ( ± 26.1) % (HCC1954), compared with the control condition (**Figure 6B**, right Y axis and **Supplementary Figure 5B**, left plot, right Y axis). When PD-L1^+^ myeloid cells were quantified within the myeloid population, this tendency to decrease was maintained in the SKBR3 co-cultures, although without statistical significance (**Figure 6C**), suggesting that this effect may be caused by a depletion of PD-L1^+^ myeloid cells and not by a specific downregulation of the ligand. These results suggest an induction of an immune effector state by the dual HER2 blockade challenge that is consistent with clinical response to trastuzumab therapy (32). Contrary to SKBR3 co-cultures (**Figure 6D**), HCC1954 co-cultures presented a significant upregulation of PD-L1 in lymphoid cells, both with single and dual antibody challenge (**Supplementary Figure 5D**).

Since TAM infiltration has already been associated to immunosuppression and poor prognosis in HER2^+^ BC patients (21, 22, 96), and given that myeloid cells appear to be directly modulated by anti-HER2 antibody challenge in our models, we looked further into the myeloid subset of immune cells. In both co-cultures, single and dual anti-HER2 blockade led to a marked reduction in the proportion of myeloid cells in culture: a 69.4 ( ± 14.8) % reduction in the SKBR3 co-cultures (**Figure 6E**, left Y axis) and 39.7 ( ± 19.7) % reduction in the HCC1954 co-cultures (**Supplementary Figure 5E**, left Y axis), with dual antibody challenge. To investigate the identity of the myeloid cells affected by this antibody challenge, we assessed expression of CD14, a general marker of monocytes and macrophages (97). In both models, challenge with trastuzumab and with the antibody combination led to a specific and significant decline in the CD14^+^ myeloid cell population (**Figure 6E**, right Y axis and **Supplementary Figure 5E**, right Y axis), suggesting a particular depletion of the monocyte/macrophage immune subset, which has been associated with clinical trastuzumab response (32). Despite the observed inter-donor variability in soluble factor secretion upon dual antibody challenge (**Supplementary Figure 7**), key soluble factors associated with myeloid cell maintenance and function such as IL-3, IL-8, CCL2 and VEGF (23, 98) were consistently detected in the culture supernatant of both experimental conditions (dual antibody challenge and no antibody) of all the analyzed biological replicates, prompting the need to further elucidate the mechanism driving this myeloid cell depletion. Overall, challenge of encapsulated 3D co-cultures of HER2^+^ BC aggregates and PBMCs with trastuzumab and pertuzumab led to an expressive reduction of the potentially immunosuppressive PD-L1^+^ myeloid cell population, in particular in the CD14^+^ monocytic population.

## Discussion

Despite the poor prognosis of HER2-OE BC and high relapse rates with targeted therapies, there is still a lack of *in vitro* human-based tools to study the dynamics of the immune response to therapy in this BC subtype and test novel strategies to improve it. In this work, we describe the development of 3D heterotypic co-cultures to model HER2-OE BC, specifically the interaction between cancer cells and the immune populations found in the tumor microenvironment. We developed what is, to the best of our knowledge, the first study of the dynamic response of distinct immune subsets of the TME to dual HER2 blockade. To address it, we developed an innovative 3D model of the HER2-OE BC immune microenvironment, generated without animal-based matrices to avoid the confounding factors of exogenous signaling modulators, and in a dynamic culture system, which allows non-destructive sampling along extended culture times.

The 3D models developed in this work are based on human cancer cell lines and human PBMCs, employing a culture strategy previously developed by our team to potentiate TME interactions between tumor cells, fibroblasts and macrophages (60, 61). This strategy takes advantage of: agitation-based cultures strategies, to improve mass and gas transfer; and cell immobilization in an inert hydrogel, to assure the proximity between cell compartments and their spatial organization, and to provide physical protection from the sheer stress of agitation-based cultures. The BC models based on 3D co-cultures of tumor and immune cells reported in literature are often dependent on functionalized or animal-based scaffolds for 3D cell growth, such as Matrigel (a complex, undefined matrix obtained from murine tumors (99)) (36, 40–43, 45, 46, 48, 49, 51–53, 55, 57, 100, 101). The scaffold-free models previously reported were maintained in static cultures (44, 46, 47, 50, 54, 56, 102), limiting culture time, assay throughput and sampling ease. By generating 3D cancer aggregates in stirred tanks or Aggrewell plates (which promote more compactness than 96-well plates due to their inverted pyramid shape (103)), we avoided the use of undefined animal-based scaffolds and promoted native-like interactions between cancer cells. Moreover, encapsulation of the co-cultures within an alginate hydrogel enables the use stirred tank-based culture systems that can be scaled up or down easily and that allow non-destructive sampling, unlike static cultures (63).

ADCC mediated by NK cells has been proposed as a fundamental mechanism of action of trastuzumab in HER2^+^ BC, based on several *in vitro* and *in vivo* studies (6, 7, 35, 62, 104, 105). Pertuzumab can also engage the NK cell activating FcγRIIIA (CD16) receptor and induce specific killing (ADCC) of the opsonized HER2^+^ tumor cells (9, 106). Remarkably, more than 20 years later, little is known about the dynamics of NK cell function in the TME of BC patients undergoing anti-HER2 blockade treatment, other than trastuzumab-based regimens induce an increase in the proportion of NK cells infiltrating the tumor mass (35). Accordingly, the most favorable clinical response has been observed in patients who retain NK cell markers in the tumor infiltrate (26) and display peripheral NK cells with higher cytotoxic potential (6, 7), dependent on CD16 expression (6, 85). With the aim of interrogating the immune effects induced by targeted therapies for BC, we optimized culture parameters to maximize the representation and functionality of the immune compartment, especially the NK cell population. IL-15 supplementation improved the representation of the original proportion of NK cells in the immune compartment at day 4 of culture, despite the overall decrease in immune cell viability. With cytokine supplementation, NK cells also exhibited a modest reduction in CD16 presentation, which suggested these cells were responsive to activating stimuli. In fact, this was corroborated in the 4-day cytotoxicity assays, where isolated NK cells mediated ADCC against HER2-OE cell aggregates in the presence of trastuzumab (**Supplementary Figure 3C**), and in the antibody challenges in encapsulated co-cultures, which led to a downregulation of CD16 expression in NK cells (**Figures 5B and C**). CD16 downregulation has been reported to occur in the CD56^dim^ CD16^+^ subset of NK cells as a consequence of NK-mediated ADCC (87, 94, 107), namely trastuzumab-induced ADCC (6). Considering the increase in apoptosis observed in the SKBR3 co-cultures upon antibody challenge (**Figure 5A**), and the proximity between tumor and immune cells in this co-culture (**Figure 3E**), it is plausible that the CD16 downregulation induced in NK cells by trastuzumab and pertuzumab challenge resulted from NK-mediated ADCC. However, this should be further supported by analysis of canonical markers of NK cytotoxicity such as CD107a, IFN-γ or granzyme B (108). Although analysis of ADCC assays has been typically performed with short time points (4h to 24h) (90, 93), we observed that prolonging the assay until 4 days resulted in evident ADCC mediated by the anti-HER2 antibodies at the same timepoint used in the challenge of encapsulated co-cultures (data not shown).

The two models of the HER2-OE BC immune microenvironment described in this work relied on tumor cell aggregates representing the same BC subtype (ER^-^ PR^-^ HER2^+^), albeit displaying distinct morphologies. HCC1954 cells aggregate into cohesive spherical structures with tight cell-cell interactions (63, 64) (**Figure 2A**); SKBR3 aggregates are irregular and loose (**Figure 2B**). This less adhesive phenotype is a recognized feature of the SKBR3 cell line, probably resulting from the low to no expression of several adhesion proteins such as cadherins, integrins and cell adhesion molecules (CAMs) (109). In fact, reports of aggregate formation for this cell line are rare and usually yield wide masses of cells clumped together rather than spherical structures, even when exogenous matrices are used (72, 74, 75, 110). Through gravity- and geometry-induced aggregation (Aggrewell technology), we obtained SKBR3 cell aggregates with adequate compactness (**Figure 2B**) to allow experimental handling and high throughput. The low level of cancer cell death in co-culture with PBMCs from different immune donors suggests that potential human leukocyte antigen (HLA) mismatch between the cancer cell lines employed and immune cells is not relevant in our models.

We observed a distinct immune responsiveness in each of the models. SKBR3 3D co-cultures displayed a higher magnitude of immune reactivity upon anti-HER2 antibody challenge, in the encapsulated cultures as well as in the 3D and 2D direct cytotoxicity assays. In 3D, due to the differences in morphology between the aggregates of SKBR3 (irregular shape and looser cell-cell connections) and HCC1954 (cohesive and spherical), the distance between PBMCs and SKBR3 cells was smaller and more consistent than between PBMCs and HCC1954 cells (**Figure 3E**). Therefore, we hypothesize that a wider surface area for contact with immune cells could favor tumor-immune crosstalk. This factor may also partially explain the fact that immune cells from none of the immune donors tested displayed significant NK cell-mediated basal cytotoxicity against HCC1954 3D aggregates compared with the negative control (only cancer cells, data not shown), while exhibiting cytotoxicity against the same cells grown in 2D (**Supplementary Figure 3C**).

Nevertheless, there are other cancer cell intrinsic factors contributing to the distinct immune responsiveness observed in both models. The HCC1954 cell population shows a heterogeneous expression of HER2, with the receptor being faintly detected in some cells and highly detected in others (**Figure 2D**). Previous reports have shown that, although both SKBR3 and HCC1954 cell lines display close to 100% of the population positive for HER2 protein, the actual HER2 protein content of HCC1954 is inferior (111, 112), which probably reflects the expression heterogeneity across HCC1954 cells. These distinct levels of HER2 receptor available for binding of trastuzumab and pertuzumab can render HCC1954 cells less susceptible to ADCC, as was also evidenced in the 3D and 2D cytotoxicity assays. Still, human breast tumors often display varying degrees of heterogeneity in HER2 expression, affecting response to therapy (113) and, as such, *in vitro* cellular models of anti-HER2 response should also reflect these phenotypes. SKBR3 cells may be inherently more susceptible to NK-mediated cytotoxicity than HCC1954 cells due to the low expression of MHC-I (65, 108, 109, 114) and B7-H3 (115, 116) proteins, involved in NK cell functional inhibition (117, 118), and high expression of NK-activating ligand DNAM-1L (108); conversely, HCC1954 cells were shown to express higher MHC-I (65, 119) and B7-H3 levels (120). Furthermore, recent findings indicate a fundamental role of HER2 glycosylation patterns for the ability to bind trastuzumab (121), with sialylation further identified as an inhibitory factor of trastuzumab function (122). In this context, it would be relevant to compare the levels of HER2 sialylation among the BC cell lines used, and test whether de-sialylation could improve the immune responsiveness to anti-HER2 blockade seen in the HCC1954-based model. While several studies also report a higher susceptibility of SKBR3 cells for ADCC (89–91), other groups have found a similar magnitude of ADCC response in both cell lines (123, 124), or even a higher susceptibility of HCC1954 cells (65). Such variability can be a reflection not only of the diversity of immune response found across human donors (84) but also of the distinct readouts used and the cross-laboratory heterogeneity of cancer cell lines (125).

Of note, our results support that the anti-tumor immune effects induced by trastuzumab and pertuzumab are independent of the trastuzumab sensitivity profile of HER2^+^ cancer cells, as seen by the immune activation and cytotoxicity induced against the trastuzumab-resistant HCC1954 cell line (**Figure 4**, **Supplementary Figures 3 and 5**) (65, 89, 91). HCC1954 cells harbor a gain-of-function mutation in the *PI3KCA* gene, leading to constitutive activation of signaling downstream of HER2 (126, 127) while maintaining a high degree of membrane HER2 expression (112, 119). As such, even though HER2-bound trastuzumab and/or pertuzumab cannot disrupt intracellular signaling in HCC1954 cells, it can still interact with CD16^+^ immune cells and induce ADCC (65, 89, 91).

The most recent clinical guidelines for HER2^+^ BC therapy recommend the combination of dual HER2 blockade, with antibodies trastuzumab and pertuzumab, and taxane-based chemotherapy as the first line treatment for metastatic cases (128) and for early-stage cases in the neoadjuvant setting (129). Still, despite the widespread use and clinical validation of this dual HER2 blockade combination, the effect in the anti-tumor immune function induced by the addition of pertuzumab has been only briefly addressed in human experimental models, such as 2D ADCC assays, and yielded conflicting results (65, 66, 92, 93, 130). Our findings, employing the two HER2-OE BC microenvironment models for dual antibody challenging, suggest a direct role of trastuzumab in inducing an anti-tumoral immune response. We observed increased apoptosis and activation of the immune response via CD16 downregulation on NK cells (**Figure 5** and **Supplementary Figure 4A**), modulation of PD-L1 expression and decline of the monocyte/macrophage compartment (**Figure 6** and **Supplementary Figure 5**). With a direct cytotoxicity assay, both NK cells and PBMCs induced ADCC in presence of the therapeutic antibodies, indicating that the qualitative increase in apoptosis induced by the antibody challenge in the model employing trastuzumab-sensitive SKBR3 cells (124) may result not only from direct HER2 signaling inhibition, e.g., leading to cell cycle arrest (131), but also from enhanced immune-mediated cytotoxicity (131).

The application of this dual-HER2 blockade also affected PD-L1 expression on the immune compartment of HER2-OE BC co-cultures. Recently, it was found that an effective anti-tumor immune response driven by trastuzumab can lead to PD-L1 upregulation on HER2^+^ cancer cells via IFN-γ release (91). Nonetheless, the effect of anti-HER2 agents over PD-L1 expression in lymphoid cells remains understudied. Available reports indicate that PD-L1 expression on tumor-infiltrating lymphocytes before the start of treatment appears to confer better survival (132, 133) and response to trastuzumab-containing therapy (28), but only one study describes downregulation of PD-L1^+^ TIL after neoadjuvant chemotherapy (NAC) with trastuzumab, with no clear association with clinical response (28). Our findings support the upregulation of PD-L1 in the lymphocyte subset of immune cells upon dual HER2 blockade. Such discrepancy can be due to the distinct sampling timepoints employed (4 days *versus* after completion of NAC (28)), capturing different phases of the immune response to HER2 blockade, but also to the distinct response abilities of healthy *versus* patient-derived immune cells. On the other hand, and in light of a recent study reporting an association between PD-L1^+^ TAM and poor response to trastuzumab in HER2^+^ BC patients (32), our observations on the marked reduction of PD-L1-expressing myeloid cells support the hypothesis of the induction of a tumor immune microenvironment favorable for clinical response and tumor regression. Since TAM and other myeloid cells display the highest immune expression of PD-L1 in the tumor microenvironment of BC (30, 134), reducing the representation of these potentially immunosuppressive cell populations is the goal of several therapeutic strategies for modulation of the tumor immune microenvironment (135). Of note, when applying this proof-of-concept to our 3D models of HER2-OE BC immune microenvironment, we observed PD-L1 modulation as early as 4 days into the antibody challenge. Given that patient-derived tumor samples are usually only obtained before treatment and/or after the end of treatment and thus can only capture the long-term effects of this therapy (28, 30, 32), our observations also highlight the importance of investigating the short- and medium-term effects of anti-HER2 therapies over the immunosuppressive state of the human TME. For this type of study, advanced 3D models can be particularly useful and may even include PBMCs from patients.

We also found evidence supporting a direct role of trastuzumab and pertuzumab in reshaping the composition of the immune microenvironment of HER2-OE BC. In both 3D co-cultures, both single and dual HER2 blockade induced a marked depletion of the myeloid compartment, including the CD14^+^ monocyte/macrophage population. The magnitude of this effect was consistent with that observed for PD-L1^+^ myeloid cell reduction, suggesting that the main phenomenon inducing the decline in PD-L1 immune positivity was in fact myeloid cell depletion. Although the mechanism driving this effect requires further elucidation, our observations do not evidence a specific depletion of soluble factors critical for myeloid cell survival upon anti-HER2 antibody challenge, as seen via cytokine antibody arrays (**Supplementary Figure 7**). Moreover, our observations are consistent with the reported dynamics of TAM depletion in the TME of HER2^+^ BC patients positively responding to trastuzumab-based therapy (32). In a pre-clinical model, this macrophage depletion was also found to correlate with improved anti-tumor response and increased infiltration of killer cells, including NK cells (32), which were previously shown to kill macrophages in distinct cancer contexts (136, 137). Similarly, a decrease of CD14^+^ monocytes was also observed in co-cultures of HER2^+^ BC cells with PBMC treated with trastuzumab and FAP-IL-2v (an engineered IL-2 variant), a therapeutic combination being evaluated in clinical trials (36). Evaluation of baseline and post-treatment samples from clinical studies could corroborate the results we obtained in the 3D models and would allow drawing associations with clinical benefit.

Importantly, we found evidence suggesting that the co-administration of pertuzumab with trastuzumab may further heighten the immune activation state at the tumor site, reducing the PD-L1^+^ immune population and enhancing the ADCC response. Similar to trastuzumab, pertuzumab is produced as an IgG1 isotype for clinical application (138), enabling the binding to FcγRIIIa (CD16) on immune cells and triggering ADCC (106). Moreover, trastuzumab and pertuzumab bind to distinct domains of the HER2 receptor and simultaneous binding can occur without competition, while maintaining protein conformation (139). As such, co-administration of both antibodies should enhance the formation of cancer-immune complexes, compared with any single agent. In this context, our findings indicate that trastuzumab and pertuzumab may act additively not only in their direct cytostatic effect, as extensively reported (140), but also in enhancing immune function at the tumor site. The lack of consensus of previous reports evaluating the synergism between trastuzumab and pertuzumab on the anti-tumor immune function (65, 66, 92, 93, 130) suggests that the impact of the dual blockade on ADCC may be dependent on the experimental model system used and on the specific composition of each patient’s tumor microenvironment. Our work builds upon previous reports (92, 93) by showing that, in a tumor microenvironment populated by several different immune subsets, pertuzumab acts additively to trastuzumab, enhancing cytotoxicity (**Figure 4**) and the depletion of immunosuppressive myeloid cells (**Figure 6** and **Supplementary Figure 5**). With the use of distinct HER2^+^ BC cell lines and PBMC from several human donors, this work attempts to better mimic patient heterogeneity, which can be further achieved by using patient-derived PBMCs or even *ex vivo* approaches with patient tumor samples (141). At the same time, this lack of consensus also highlights the relevance of defining standard disease modeling technologies, readouts, and assay parameters to evaluate *in vitro* the immune effects of novel and current cancer therapies.

Overall, the 3D models of HER2-OE BC immune microenvironment described in this work hold the potential to be used for interrogation of short-to-medium term effects of anti-HER2 therapies on the local immune response, capturing the initial phase of response to anti-HER2 therapies before the onset of immunosuppression, namely in the modulation of NK cell phenotype and in myeloid cell populations. As such, this experimental strategy can also potentially be adapted to represent the immune microenvironment of other solid tumors which may benefit from immunotherapeutic strategies tackling innate immune function, including therapeutic antibodies inducing ADCC. Moreover, by evidencing tumor-driven differences in immune response, these models can be useful to capture the diversity of tumor response against novel anti-HER2 agents, such as the recently approved monoclonal antibody margetuximab (142, 143) and the antibody-drug conjugate trastuzumab-deruxtecan (144).

## Data availability statement

The original contributions presented in the study are included in the article/**Supplementary Material**. Further inquiries can be directed to the corresponding author.

## Author contributions

SB: Conceptualization, Investigation, Writing – original draft, Writing – review & editing, Formal Analysis, Methodology. CMG: Writing – review & editing, Investigation, Visualization. CB: Conceptualization, Funding acquisition, Methodology, Supervision, Writing – review & editing, Data curation, Project administration.
