# Supplementary material for: Immune microenvironment dynamics of HER2 overexpressing breast cancer under dual anti-HER2 blockade
Source: Front Immunol. 2023 Oct 31;14:1267621. doi: 10.3389/fimmu.2023.1267621 (PMC10643871; doi:10.3389/fimmu.2023.1267621)
Supplement: Supplementary file 1 [file DataSheet_1.pdf]

Supplementary Material

Immune microenvironment dynamics of HER2 overexpressing breast cancer under dual anti-HER2 blockade

Sofia Batalha, Catarina Monteiro Gomes, Catarina Brito*

*** Correspondence:** Corresponding Author: anabrito@ibet.pt

Supplementary Figures

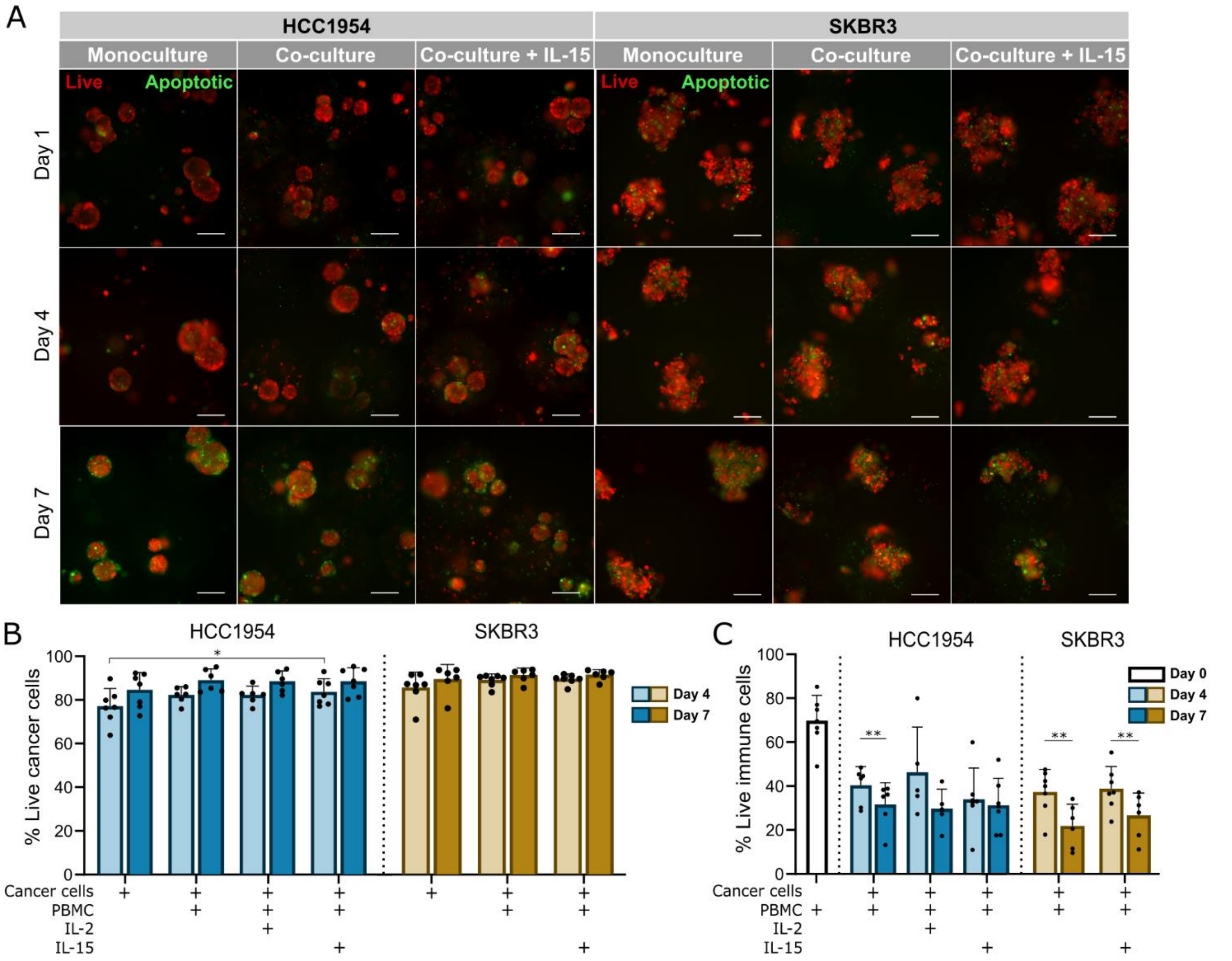

Supplementary Figure 1. Cell viability assessment of the 3D models of the immune microenvironment of HER2-OE BC.

A) Viability assessment with the fluorescent probes Mitoview (live cells, red) and Nucview (apoptotic cells, green) suggests an increase in apoptotic cells from day 4 to day 7, scattered throughout the aggregates in both monocultures and co-cultures with PBMCs of HCC1954 (left) or SKBR3 (right) and PBMCs, regardless of IL-15 supplementation. Representative pictures depicting one of 6 independent experiments, performed with different immune donors. Scale bars: 150 µm.

B) Assessment of the percentage of viable cancer cells ($CD45^{-}$ $DAPI^{-}$) at day 4 (lighter shade) and 7 (darker shade) of co-culture, by flow cytometry, indicated a high cancer cell viability throughout culture time and across conditions in both HCC1954 (blue) and SKBR3 (yellow) mono- and co-cultures with PBMCs.

C) Assessment of the percentage of viable immune cells ($CD45^{+}$ $DAPI^{-}$) before encapsulation (Day 0, white) and at day 4 (lighter shade) and 7 (darker shade) of HCC1954 (blue) and SKBR3 (yellow) co-

cultures with PBMCs, by flow cytometry, revealed a significant decline in the immune cell population from day 4 to day 7 of culture in both co-cultures.

B and C: Cell populations were identified and quantified according to the gating strategy supplied in D). Bars represent mean ± S.D. from N = 6 independent experiments, performed with different immune donors. Pairwise statistical comparisons between indicated groups performed with unpaired t-test; *, $p<0.05$; **, $p<0.01$.

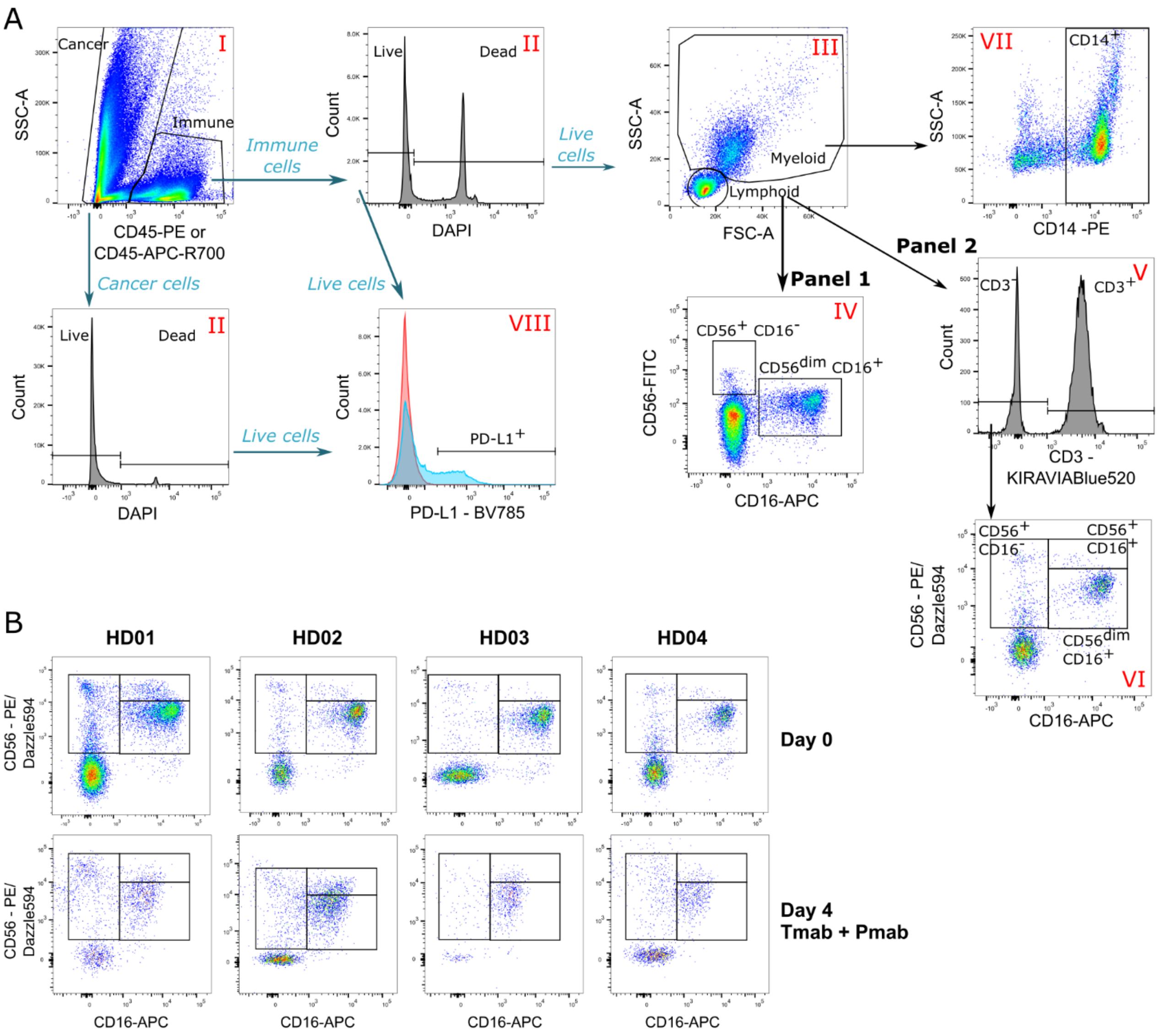

Supplementary Figure 2. Flow cytometry gating strategy.

A) Gating strategy used in flow cytometry analysis of cancer and immune cells. Cancer and immune cells were gated on the basis of CD45 expression (I), and then dead ($DAPI^+$) cells were removed from each population before further analysis (II). For immune ($CD45^+$) cells, lymphocytic and monocytic cells were separated based on size and complexity (forward scatter and side scatter) (III). Lymphocytes were either directly analyzed for the expression of NK cell markers (IV) (Panel 1) or split into $CD3^+$ and $CD3^–$ populations (V) (Panel 2). Using Panel 2, $CD3^–$ lymphocytes were analyzed for the quantification of different NK cell subsets (VI). In both panels, NK cells were identified within the lymphocyte gate by positive expression of CD56 ($CD56^+$) (either dim or bright) (IV and VI). Myeloid cells were further analyzed for the expression of CD14 (VII). Quantification of $PD\text{-}L1^+$ cells was performed after removing dead cells from $CD45^+$ or $CD45^–$ populations, and according to gate drawn

using a Fluorescence-Minus-One control (red peak on PD-L1 plot) (VIII). Panel 1 was used for Figure 3 and Panel 2 was used for Figures 5 and 6 and Supp. Fig. 4, 5 and 6.

B) NK cell gating (stage VI of the gating strategy) for four representative biological replicates of dual anti-HER2 antibody challenge on encapsulated co-cultures of SKBR3 and PBMCs (quantified in Fig. 5), depicting NK cell populations before co-culture (day 0, upper row) and after 4 days of antibody challenge (day 4, bottom row). The 4-day dual anti-HER2 antibody challenge induced a modulation of the NK cell compartment. Co-cultures were supplemented with IL-15.

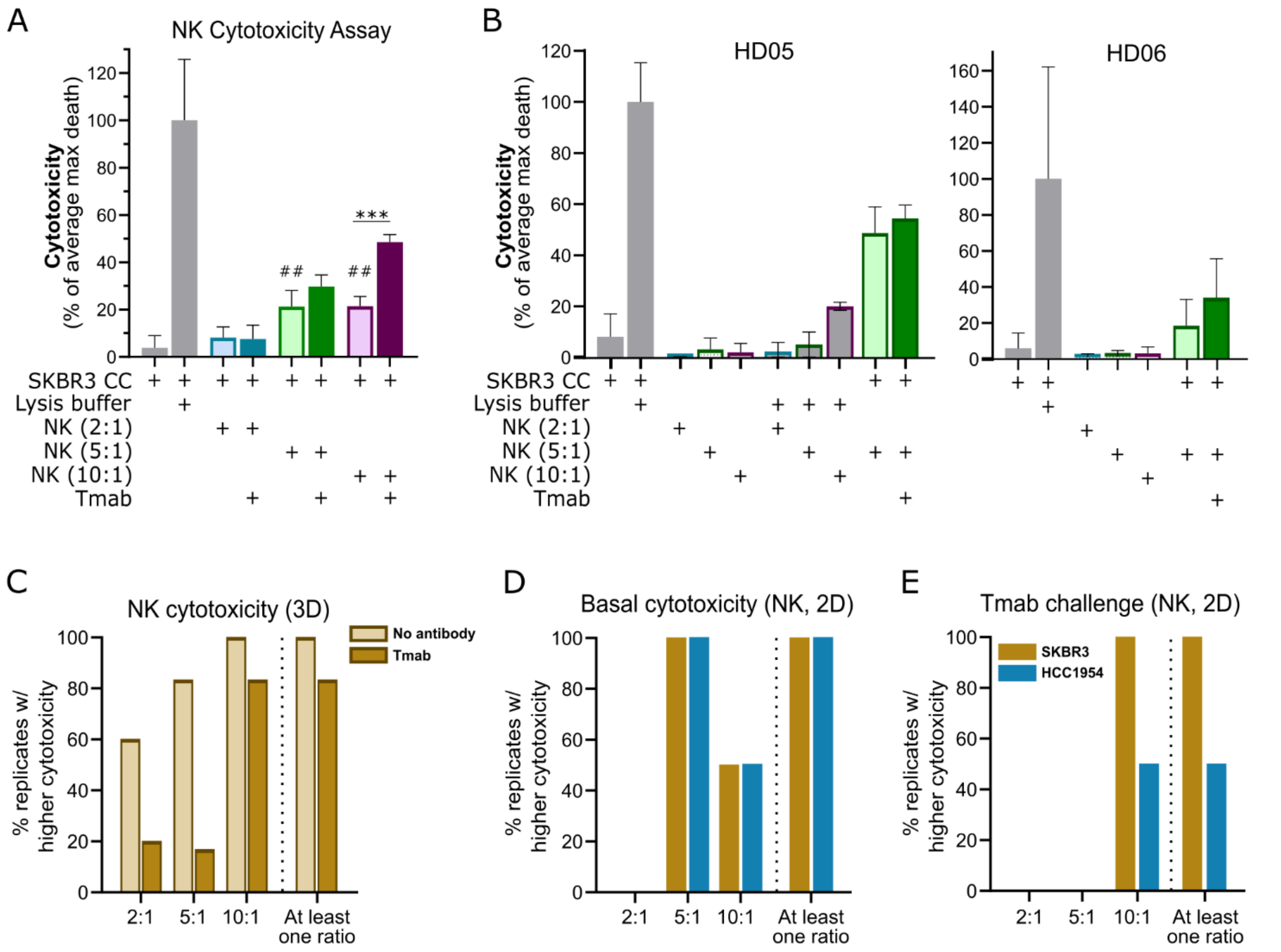

Supplementary Figure 3. Single anti-HER2 antibody challenge can induce ADCC against aggregates of HER2-OE breast cancer cell lines via NK cells

A) Direct cytotoxicity assay performed with SKBR3 aggregates and NK cells at different effector (NK) : target (cancer cell) ratios (E:T; 2:1, blue; 5:1, green; 10:1, purple), by assessment of LDH leakage after 4 days of challenge. Tested conditions included negative (cancer cells only) control; positive (cancer cells with lysis buffer) control, to determine maximal cancer cell death (max death); no challenge and single (trastuzumab, Tmab) antibody challenge, assayed at the different E:T. Data from one experiment representative of those depicted in (C); bars represent mean ± S.D. of five technical replicates. Pairwise statistical comparisons between indicated groups (*) or between the indicated condition and negative control (#) performed with unpaired t-test: ##, $p<0.01$, ***, $p<0.001$.

B) Assessment of LDH leakage derived from spontaneous or maximum NK cell death in the context of the direct cytotoxicity assay described in (A) indicates that the contribution of NK cell death to the final assay readout is residual. Bars represent mean ± S.D. of five technical replicates.

C) Assessment of the percentage of biological replicates in which SKBR3 cell death (measured by LDH leakage) was significantly higher in direct co-culture of NK cells and SKBR3 cancer cell aggregates, with or without antibody challenge, at different E:T ratios demonstrated that NK cell basal

cytotoxic ability (lighter shade) could be boosted in presence of Tmab (darker shade). Percentage of biological replicates in which tumor cell death was significantly ($p<0.05$) higher: in the presence of NK than in negative control with only cancer cells (no antibody) or in the presence of NK + Tmab than in the presence of NK cells only (Tmab). The percentage of immune cells from distinct donors that responded in at least one of the ratios tested (At least one ratio) is also plotted. NK cells from 6 different donors were assayed independently.

D) Assessment of the percentage of biological replicates in which SKBR3 (yellow) or HCC1954 (blue) cell death (measured by LDH leakage after 4 days of challenge in 2D co-culture), at the indicated E:T ratios, was significantly ($p<0.05$) higher in the presence of NK cells than negative control with only cancer cells, revealed that NK cells exhibit basal cytotoxicity against both $HER2^+$ breast cancer cell lines in 2D. The percentage of biological replicates displaying increased cytotoxicity in at least one of the ratios tested (At least one ratio) is also plotted. Plot indicates percentage of biological replicates in which tumor cell death was significantly ($p<0.05$) higher in in the presence of NK cells than negative control with only cancer cells, evaluated by unpaired t-test for each of the E:T ratios, for each of the cell lines. NK cells from 2 different donors, assayed independently.

E) Assessment of the percentage of biological replicates in which SKBR3 (yellow) or HCC1954 (blue) cell death (measured by LDH leakage after 4 days of challenge in 2D), at the indicated E:T ratios, was significantly ($p<0.05$) higher in the presence of NK cells + Tmab than in the presence of NK cells only demonstrated that HCC1954 cells are less susceptible than SKBR3 to trastuzumab-induced, NK-mediated ADCC in 2D. The percentage of biological replicates displaying increased cytotoxicity in at least one of the ratios tested (At least one ratio) is also plotted. Plot indicates percentage of biological replicates in which tumor cell death was significantly ($p<0.05$) higher in the single antibody challenge than without antibody, evaluated by unpaired t-test for each of the E:T ratios, for each of the cell lines. NK cells from 2 different donors, assayed independently.

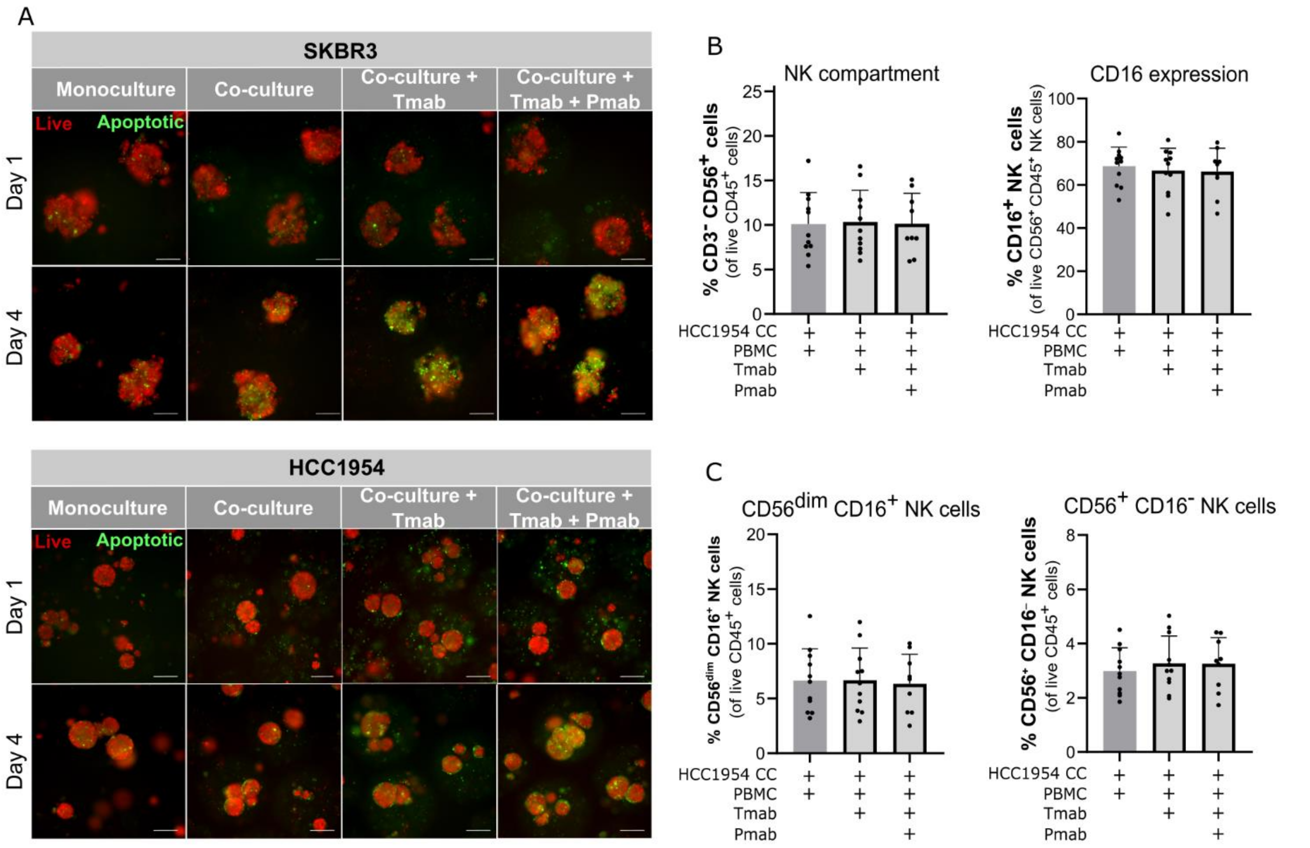

Supplementary Figure 4. HCC1954-based 3D model of the immune microenvironment of HER2-OE BC challenged with anti-HER2 antibodies for 4 days displays increased cell death but no modulation of the NK cell population

A) Viability assessment with the fluorescent probes Mitoview (live cells, red) and Nucview (apoptotic cells, green) revealed an increase in apoptosis in SKBR3 (top panel) and HCC1954 (bottom panel) aggregates upon 4 days of single (trastuzumab, Tmab) or dual (trastuzumab plus pertuzumab, Tmab + Pmab) anti-epidermal growth factor receptor 2 (HER2) antibody challenge. Co-cultures were supplemented with IL-15. Representative pictures depicting one of 9 independent experiments, performed with different immune donors. Scale bars: 150 μm.

B) Percentage of NK cells ($CD3^{-}CD56^{+}$) and $CD16^{+}$ NK cells ($CD3^{-}CD56^{+}CD16^{+}$) within the viable immune cell population ($CD45^{+}$ $DAPI^{-}$), detected by flow cytometry. The representativity of NK cells in the immune population and the $CD16^{+}$ NK subset were not impacted by the 4-day single and dual anti-HER2 antibody challenges. Co-cultures were supplemented with IL-15.

C) Percentage of the two major NK cell subsets ($CD3^{-}CD56^{dim}$ $CD16^{+}$ and $CD3^{-}CD56^{+}CD16^{-}$) in the viable immune cell population ($CD45^{+}DAPI^{-}$), detected by flow cytometry. Neither of the NK cell subsets were impacted by the 4-day single and dual anti-HER2 antibody challenges. Co-cultures were supplemented with IL-15.

B and C: Cell populations were identified and quantified according to gating strategy supplied in Supp. Fig. 1D (Panel 2). Bars represent mean ± S.D. of $N \geq 9$ independent experiments, performed with

different immune donors. Pairwise statistical comparisons, relative to co-cultures with IL-15 and without antibody challenge, evaluated by a paired t-test: all comparisons are non-significant ($p>0.05$).

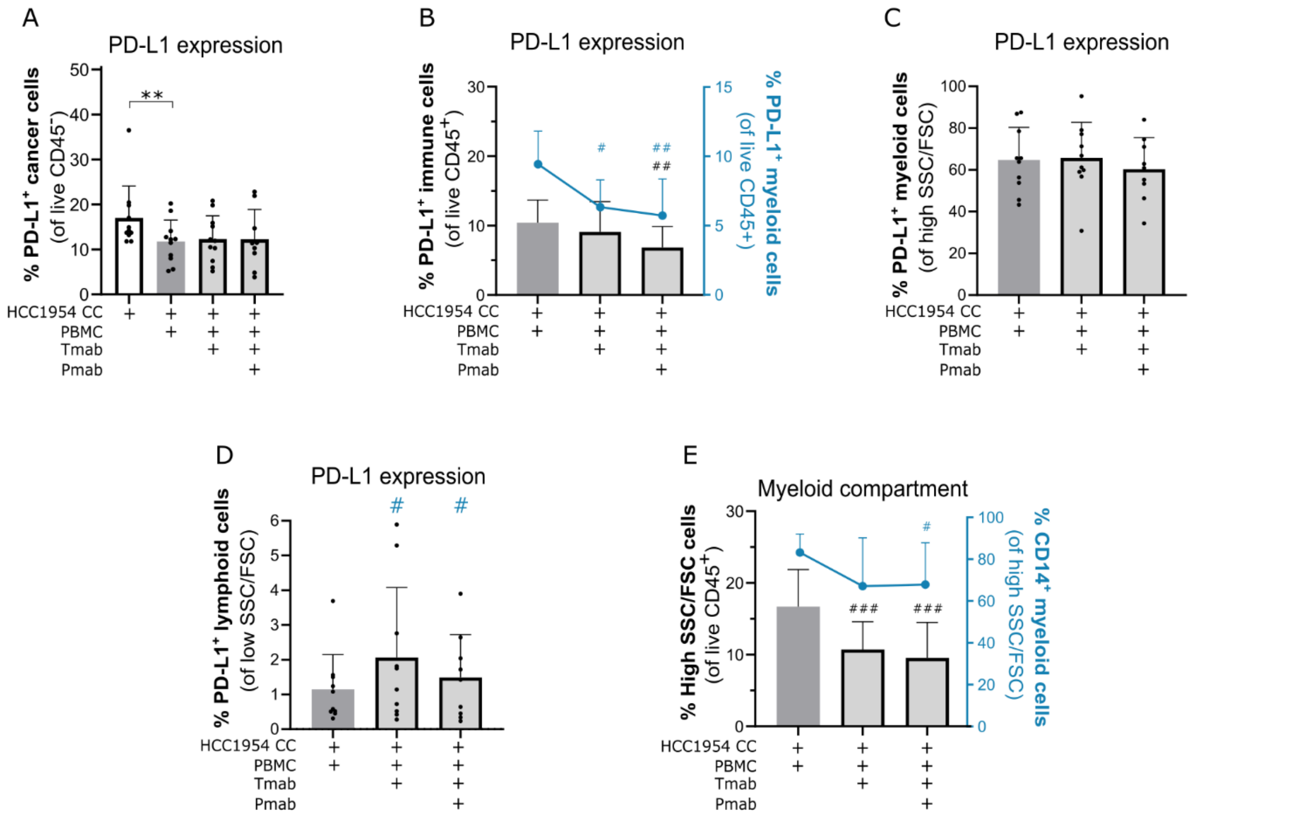

Supplementary Figure 5. Challenge of HCC1954-based 3D model of the immune microenvironment of HER2-OE BC with anti-HER2 antibodies led to downmodulation of PD-L1 in myeloid cells and a decline in the myeloid compartment.

A) Assessment of the percentage of programmed death-ligand 1 (PD-L1)$^{+}$ cancer cells within all viable cancer cells (CD45^{-} DAPI^{-} cells), by flow cytometry, showed that single (trastuzumab, Tmab) and dual (trastuzumab plus pertuzumab, Tmab + Pmab) anti-epidermal growth factor receptor 2 (HER2) antibody challenge did not significantly affect expression of the PD-L1 checkpoint ligand in cancer cells, but co-culture with PBMC induced a downregulation of PD-L1 in cancer cells compared with the monoculture, at day 4, in the HCC1954 co-culture model supplemented with IL-15.

B) Percentage of PD-L1^{+} immune cells within all viable immune cells (CD45^{-} DAPI^{-}, left plot, left Y axis, black) and percentage of PD-L1^{+} live myeloid cells (PD-L1^{+} SSC^{hi}/FSC^{hi} in the total CD45^{+} DAPI^{-} viable immune cell population, left plot, right Y axis, blue), detected by flow cytometry. The 4-day single and dual anti-HER2 antibody challenge induced a reduction of PD-L1 expression in the global immune population, specifically in the myeloid compartment. Co-cultures supplemented with IL-15.

C) Percentage of PD-L1^{+} live myeloid cells (PD-L1^{+} in the total SSC^{hi}/FSC^{hi} CD45^{+} DAPI^{-} live myeloid cells), detected by flow cytometry. There was no apparent tendency to downregulate PD-L1

expression in the myeloid population upon 4 days of single and dual anti-HER2 antibody challenge. Co-cultures supplemented with IL-15.

D) Percentage of PD-L1$^+$ live lymphoid cells (PD-L1$^+$ in the total SSC^{low}/FSC^{low} lymphoid cell population), detected by flow cytometry. The 4-day single and dual anti-HER2 antibody challenge induced an upregulation of PD-L1 in the lymphoid compartment. Co-cultures supplemented with IL-15.

E) Percentage of live myeloid cells (SSC^{hi}/FSC^{hi} in the CD45$^+$ DAPI$^-$ total viable immune cell population, left Y axis, black) and the percentage of CD14$^+$ monocytic cells (CD14$^+$ in the total SSC^{hi}/FSC^{hi} CD45$^+$ DAPI$^-$ live myeloid cells, right Y axis, blue), detected by flow cytometry. The 4-day single and dual anti-HER2 antibody challenge induced a decline in the proportion of myeloid cells within the immune compartment, particularly the CD14$^+$ monocytic cells. Co-cultures supplemented with IL-15.

A, B, C, D and E: Cell populations were identified and quantified according to gating strategy supplied in Supp. Fig. 2A (Panel 2). Bars represent mean ± S.D. of N = 5 (quantification of CD14$^+$ SSC^{hi}/FSC^{hi} CD45$^+$ DAPI$^-$) and N = 9 (all other quantifications) independent experiments, performed with different immune donors. Pairwise statistical comparisons, relative to co-culture with IL-15 and without antibody challenge (#) or between indicated groups (*), evaluated with a paired t-test: #, $p<0.05$; ##, **, $p<0.01$; ###, $p<0.001$. B and E: plots displaying individual data points are provided in Supp. Fig. 6.

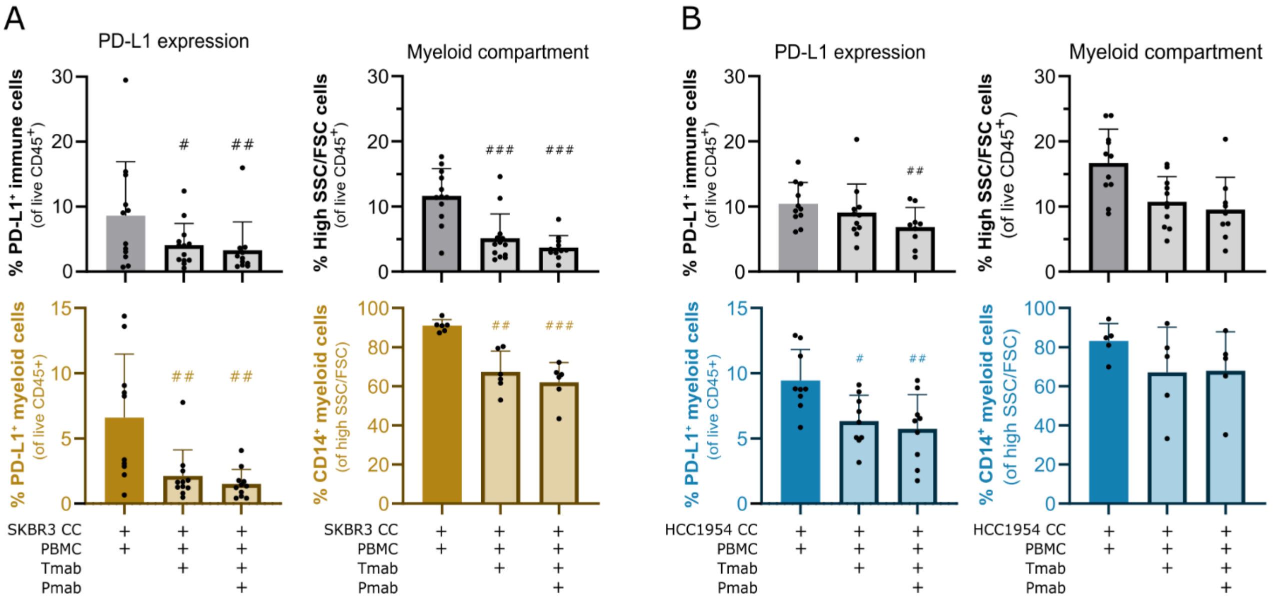

Supplementary Figure 6. Modulation of PD-L1 expression and of the myeloid compartment upon single and dual anti-HER2 challenge in SKBR3- and HCC1954-based 3D models.

A) Representation of the results of SKBR3-based 3D model antibody challenge displayed in Fig.6 B (left column) and E (right column) as individual datapoints, each corresponding to an independent biological replicate.

B) Representation of the results of HCC1954-based 3D model antibody challenge displayed in Supp.Fig.5 B (left column) and E (right column) as individual datapoints, each corresponding to an independent biological replicate.

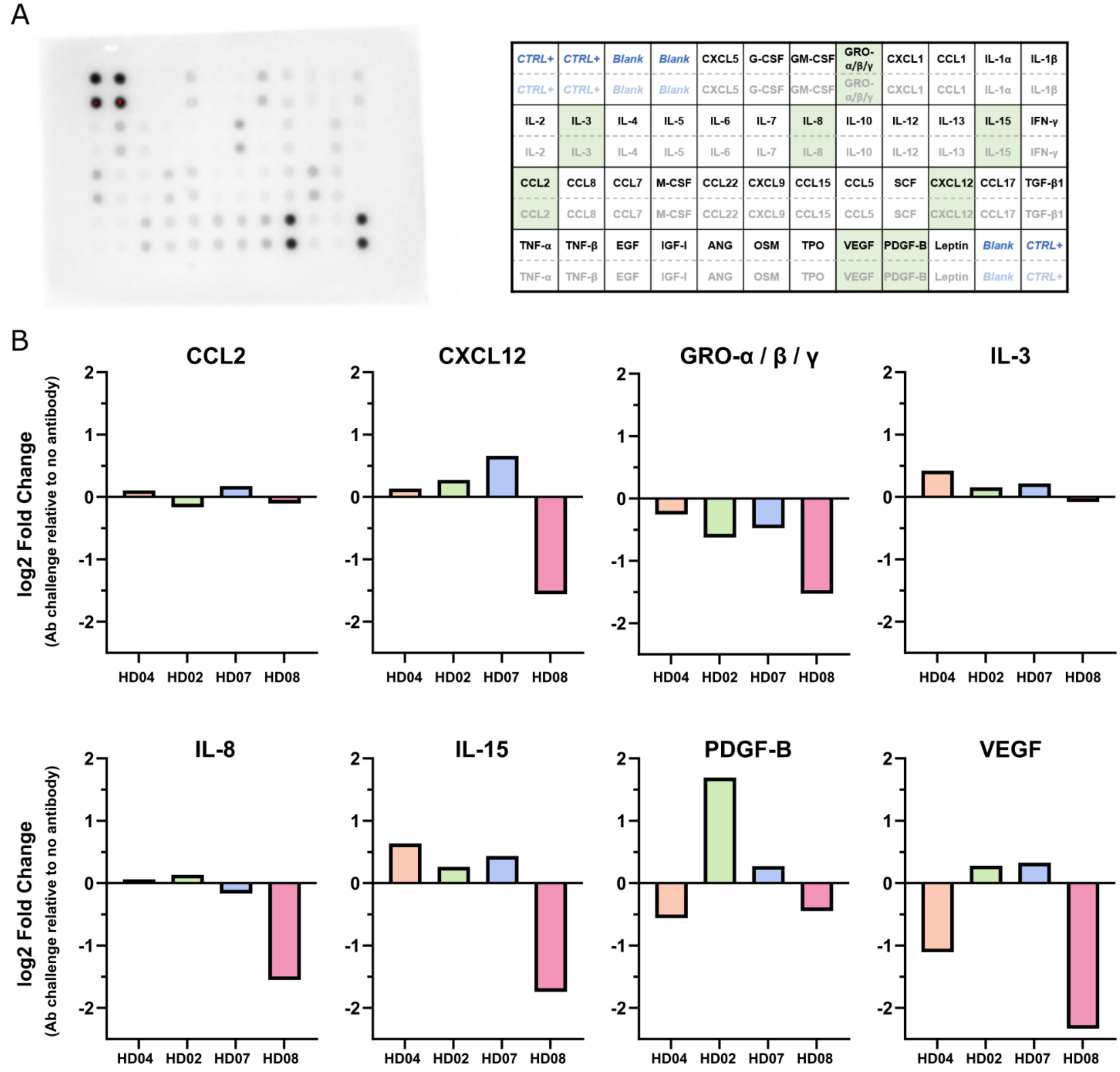

Supplementary Figure 7. Soluble factors associated with immune function are present in the supernatant of SKBR3-based 3D model of the immune microenvironment of HER2-OE BC, with and without challenge with anti-HER2 antibodies.

A) Chemiluminescent detection of cytokines and chemokines present in the supernatant of the SKBR3-based 3D model of the immune microenvironment of HER2-OE BC, with and without dual anti-HER2 antibody challenge, was performed using a commercially available human cytokine antibody array. Left: representative image of a membrane used for chemiluminescent detection (HD07, dual antibody challenge, 5 sec exposure). Right: array map displaying the position of each cytokine detection spot on the membranes (same orientation as the membrane depicted). Soluble

factors detected in all experimental replicates and conditions tested are highlighted in green shade. ANG: angiogenin; OSM: oncostatin M; TPO: thrombopoietin.

B) Semi-quantitative chemiluminescent detection of cytokines and chemokines in the supernatant of this model, represented as log2-transformed fold change of the “dual antibody challenge” over “no antibody”, reveals heterogeneous soluble factor secretion phenotypes across distinct PBMC donors. N = 4 independent experiments, performed with different immune donors.
